# Design Strategies Toward Zinc Anodes with High Utilization Rate for Practical Aqueous Zinc-Ion Batteries

**DOI:** 10.1007/s40820-026-02301-w

**Published:** 2026-07-20

**Authors:** Yahan Meng, Jintao Qi, Apeng Li, Xiang Li, Ze Xu, Kunjie Ding, Mingming Wang, Ying Ian Chen, Shaoming Huang

**Affiliations:** 1 School of Chemistry and Materials Science, Hangzhou Institute for Advanced Study, University of Chinese Academy of Sciences, Hangzhou, 310024 People’s Republic of China; 2 Institute for Frontier Materials, Deakin University, Melbourne, Australia

**Keywords:** Aqueous Zn batteries, Zn anode, High Zn utilization rate, Large-scale energy storage

## Abstract

Aqueous zinc-ion batteries (AZIBs) have emerged as promising candidates for large-scale energy storage systems due to their high safety, low cost, and environmental friendliness. However, the zinc (Zn) anode faces a series of side reactions, including hydrogen evolution, dendrite growth, corrosion, and passivation, leading to irreversible loss of active Zn material and a significant reduction of cycling stability of the Zn anode. To mitigate the impact of these issues, an excess of Zn anode is commonly employed to ensure a continuous supply of Zn during long-term operation. However, the use of excess Zn results in a practical energy density of AZIBs that is far below the requirements for commercialization. Improving Zn anode utilization rate (ZUR) and optimizing the negative/positive electrode capacity ratio (*N*/*P*) are effective pathways to achieve high energy density. This review systematically summarizes the challenges associated with Zn anodes with high ZUR and provides a detailed discussion on modification strategies to improve the ZUR from three aspects: the anode, electrolyte, and separator. Finally, we look ahead to the future development directions and prospects of Zn anodes with high ZUR and AZIBs with high energy density. With ongoing technological advancements and continuous innovation, we believe AZIBs have the potential to overcome current bottlenecks and contribute to the global development of sustainable energy systems.

## Highlights

- *ZUR* *as Key Metric:* High zinc utilization rate (ZUR) is identified as the decisive factor for bridging the gap between theoretical and practical energy densities in aqueous zinc-ion batteries (AZIBs).
- *Tri-part Modification:* The review systematically evaluates current advancements in stabilizing zinc anodes under high ZUR by engineering the anode structure, electrolyte composition, and separator functions.
- *Commercial Roadmap:* Future perspectives emphasize standardized testing—including low N/P ratios and lean electrolytes, alongside AI-driven material screening to accelerate AZIB industrialization.

## Graphical abstract

**Figure:**
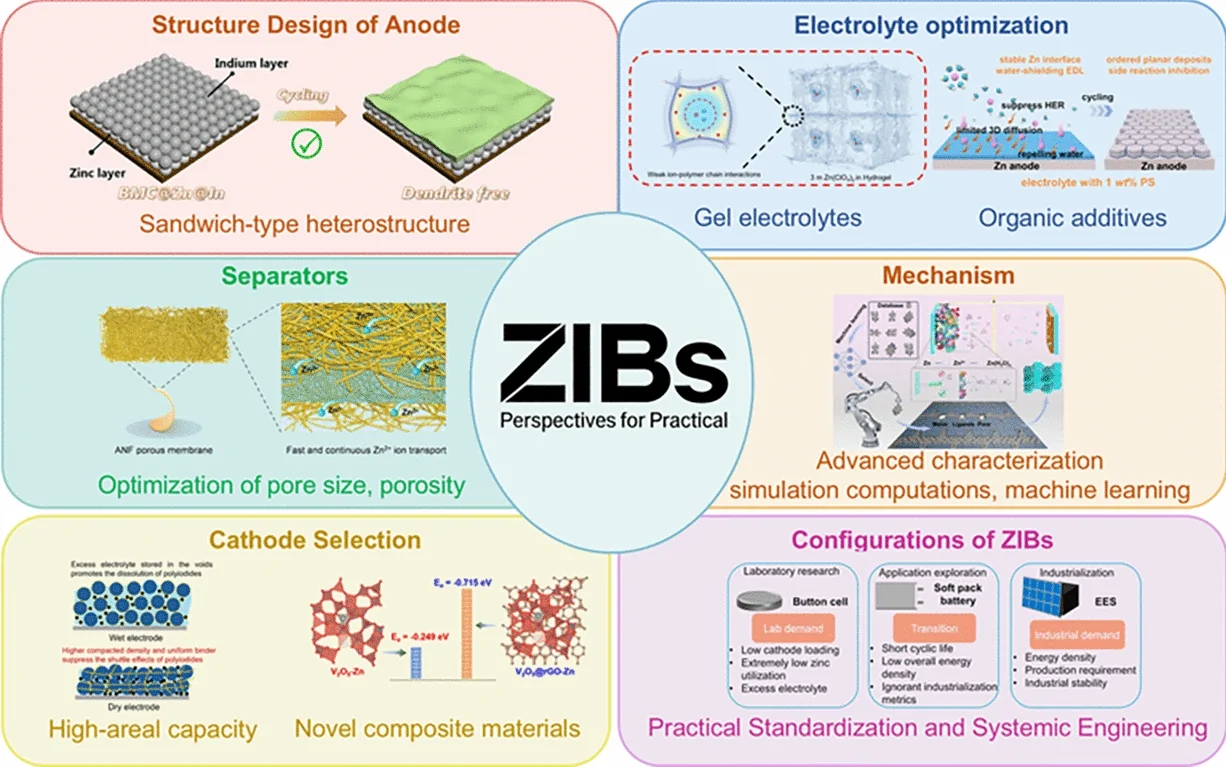

## Introduction

The utilization of renewable energy is crucial for alleviating the global energy crisis and environmental pollution [1, 2]. However, the intermittency and discontinuity of renewable energy sources such as wind and solar power necessitate the development of energy storage technologies that can match their generation characteristics [3, 4]. Among these, electrochemical energy storage technologies, particularly rechargeable batteries, are widely employed in portable electronic devices, electric vehicles, and grid-scale energy storage due to their high efficiency and flexibility [5, 6].

Currently, while commercial lithium-ion batteries (LIBs) dominate the markets for electric vehicles and portable electronic devices, the relatively high cost of lithium resources and safety issues associated with organic electrolytes make it essential to explore safer and more cost-effective alternative battery technologies [7–10]. Such alternatives are anticipated to serve as substitutes for LIBs in specific application areas such as the large-scale energy storage field [11]. Compared to organic electrolyte-based LIBs, aqueous batteries are considered a promising energy storage technology for grid storage due to the inherent safety of their aqueous electrolytes [12–17]. In particular, aqueous zinc-ion batteries (ZIBs) have attracted significant research and industrial interest due to their safety, low cost, non-toxicity, ease of manufacturing, and fast charge/discharge capabilities [18–26]. For energy storage solutions requiring low cost and high safety, ZIBs have potential applications in large-scale energy storage systems, particularly replacing LIBs and flow batteries in renewable energy storage, grid regulation, as well as in lead-acid batteries in electric vehicles [27–32].

However, in traditional ZIBs, the unstable anode/electrolyte interface during the Zn plating/stripping process inevitably leads to side reactions such as hydrogen evolution reaction (HER) [33], corrosion [34], passivation [35], and dendrite growth [36], resulting in low Coulombic efficiency (CE), poor cycling stability, and even short circuit of the Zn anode [37–40]. In addition to these issues, the Zn utilization rates (ZURs) is often severely underestimated. A key issue in current research is that, to compensate for the irreversible loss of Zn and enhance the cycling stability during charge/discharge processes, researchers typically use excess Zn anodes, which leads to a low ZUR (typically less than 10%), thereby limiting the practical energy density of ZIBs.

**Fig. 1:**
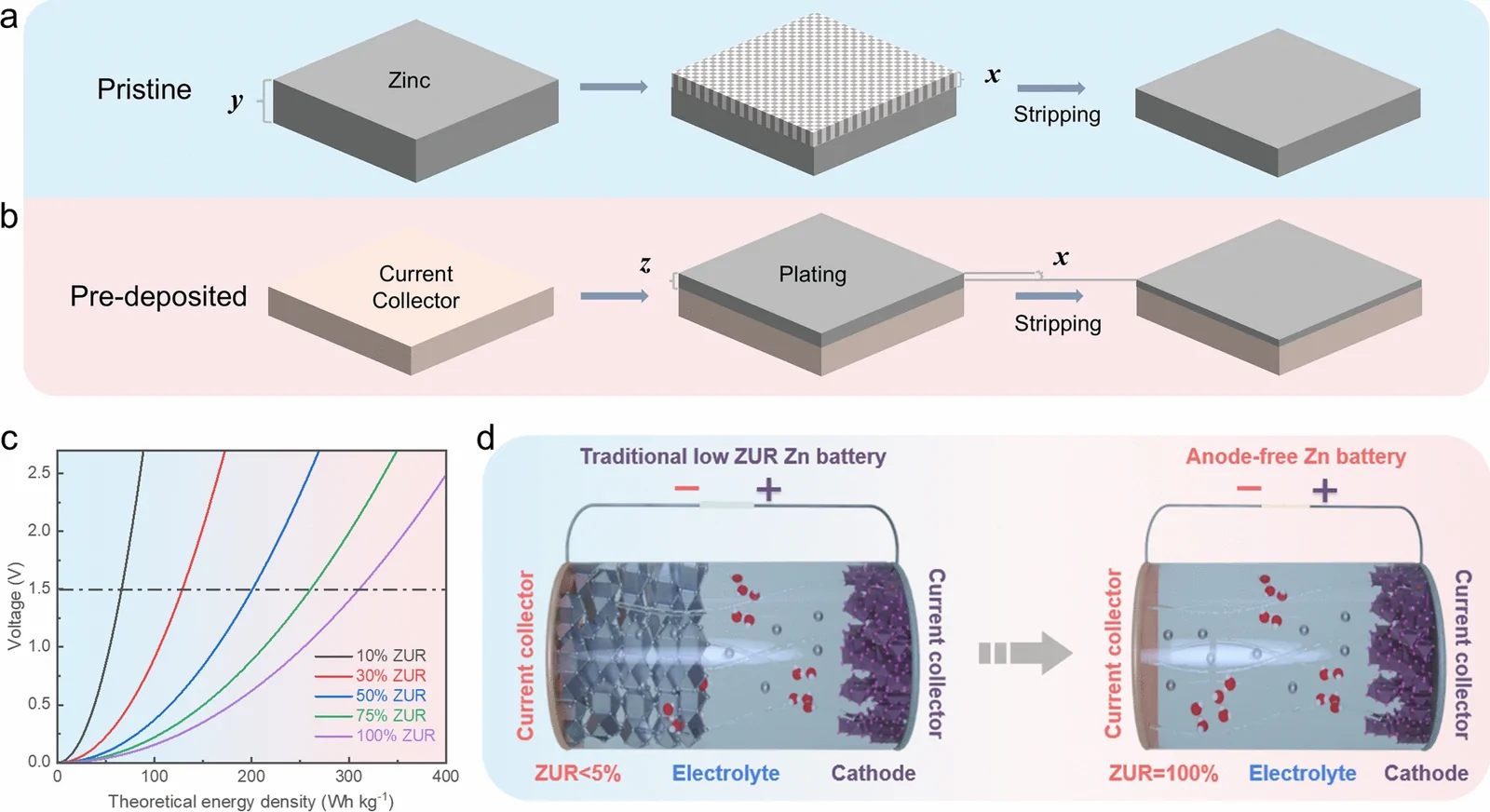
Schematic illustrating the definition and significance of ZUR. **a** ZUR of pristine Zn anode. **b** ZUR of pre-deposited Zn anode. **c** Theoretical energy density of AZIBs at different ZURs and discharge voltages, where the theoretical specific capacity of the cathode is set at 200 mAh g^−1^. Copyright 2025, Elsevier. **d** Comparison of the structures of conventional Zn batteries and anode-free Zn batteries. From Ref. [10]. Copyright 2025, Elsevier

First, it is necessary to define ZUR. The anodes used in current ZIBs can be categorized into pristine Zn anodes and pre-deposited Zn anodes. The pristine Zn anode refers to using metallic Zn foil or Zn powder directly as the anode (Fig. 1a). Under these conditions, ZUR can be calculated based on the actual cycling capacity (*x* mAh) and theoretical capacity (*y* mAh), where the theoretical capacity is determined by the mass of the Zn source (*m* g) and the theoretical specific capacity of Zn (820 mAh g^−1^). The formula used to calculate the ZUR for a pristine Zn anode is as follows:

1 $$\mathrm{Z}\mathrm{U}\mathrm{R}=\frac{x}{y}\times 100\%=\frac{x}{m\times 820}\times 100\%$$

As shown in Fig. 1b, a pre-deposited Zn anode refers to an anode formed by pre-depositing a certain amount of Zn onto a substrate that does not contain Zn as the anode for the ZIBs. Assuming the capacity of the pre-deposited Zn is *z* mAh, in this case, the formula used to calculate the ZUR is as follows:

2 $$\mathrm{Z}\mathrm{U}\mathrm{R}=\frac{x}{z}\times 100\%$$

High ZUR is of significant importance for the performance and practical application of ZIBs [41, 42]. Firstly, the ZUR directly impacts the energy density of ZIBs. A higher ZUR means that more Zn material can participate in the charge/discharge processes, thereby providing greater energy storage, which is crucial for the overall energy density of the ZIBs [43]. Assuming the theoretical specific capacity of the cathode in ZIBs is 200 mAh g^−1^, under the same discharge voltage, the higher the ZUR, the higher the theoretical energy density (Fig. 1c). For example, when the average discharge voltage of the ZIBs is 1.5 V, and assuming the anode ZUR is 10%, the theoretical energy density of the entire ZIB would be approximately 67 Wh kg^−1^. In contrast, when the ZUR is 100%, the theoretical energy density of the entire battery can reach up to 315 Wh kg^−1^ under the same condition. However, the ZUR of traditional ZIBs is typically less than 5%, which results in a significantly low energy density for the entire battery. In other words, the aqueous Zn metal anode currently limits the upper and lower performance boundaries of aqueous ZIBs. Therefore, from a practical standpoint, it is imperative to enhance the anode performance under high-ZUR conditions, enabling the development of high-energy–density ZIBs with “anode-less” or “anode-free” designs (Fig. 1d). It should be emphasized that for Zn anode-limited batteries under high-utilization conditions, the zinc utilization rate (ZUR) is numerically equivalent to the depth of discharge (DOD). In such systems, the overall battery discharge capacity is dominated by the reversible utilization of the Zn anode, enabling the two terms to be used interchangeably throughout this review.

Although improving the ZUR is crucial for enhancing the energy density of ZIBs, achieving long-cycle stability at high ZUR still faces numerous challenges [10, 44]. As previously mentioned, side reactions such as dendrites growth, HER [45], corrosion, and passivation are common during the cycling of Zn anodes, all of which negatively impact their electrochemical performance [44, 46, 47]. However, it is noteworthy that many aqueous Zn metal anodes reported in the literature maintain good stability even after thousands of hours during cycling tests. This is primarily due to the fact that the excess Zn source effectively masks or even neglects the negative effects of these side reactions under conditions of low ZUR. However, when designing Zn anodes with high ZURs, the impact of these side reactions becomes exceptionally pronounced, making it difficult for the battery to maintain stable performance under high energy density and long cycle life [48–51].

**Fig. 2:**
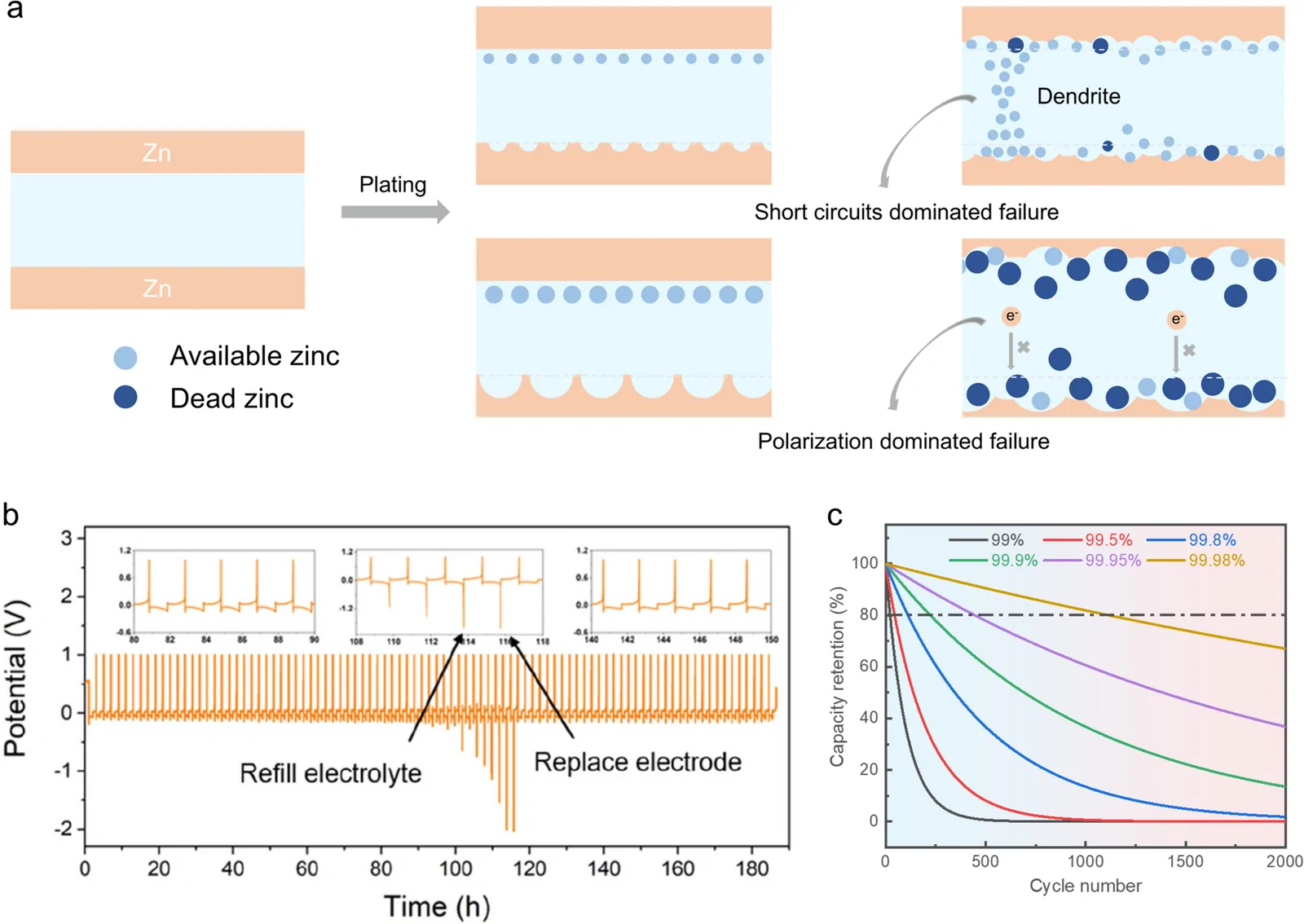
Failure mechanisms of the Zn anode at high ZUR and the correlation with CE and cycling stability. **a** Schematic illustration of Zn anode failure under high ZUR. **b** Voltage–time profile of Zn plating/stripping in the Zn–Ti cell before and after refilling the electrolyte and replacing the Zn electrode. From Ref. [54]. Copyright 2024, American Chemical Society. **c** Predicted capacity retention of anode-free Zn batteries under different CEs (assuming the battery system’s ZUR is 100%). From Ref. [10]. Copyright 2025, Elsevier

Under high-ZUR conditions, the failure mechanism of the Zn anode differs significantly from that under low-ZUR conditions [50, 52, 53]. Studies indicate that failure is typically not solely caused by short circuits due to Zn dendrite growth. Since Zn deposition/dissolution is not completely reversible, the failure under high ZUR is primarily attributed to the complete consumption of the Zn anode during repeated deposition/dissolution cycles, which leads to the overall structural degradation of the electrode [54]. Ultimately, the active regions of the electrode can no longer sustain electrochemical reactions, resulting in rapid performance decay or complete failure of the battery (Fig. 2a). For example, after a Zn(10 μm)–Ti cell cycled for approximately 112 h exhibited initial polarization failure, the cell was disassembled, reassembled with an additional 50 μL of electrolyte. When the cell was cycled again, the polarization did not improve. However, immediately replacing the cycled Zn electrode with a new one instantly eliminated the polarization issue (Fig. 2b) [54]. These results indicate that Zn depletion plays a decisive role in battery failure, particularly at high ZUR and high electrolyte/capacity ratios. Mechanistically, this polarization-dominated failure is driven by the dynamic evolution of local current density distribution and the synergistic amplification of kinetic overpotentials. Under high ZUR, the limited active Zn undergoes heterogeneous dissolution and deposition during repeated plating/stripping cycles, accompanied by the gradual deactivation of zincophilic sites and progressive interfacial degradation. This causes the local current density to evolve from an initially uniform distribution to severe spatial concentration, forming a destructive positive feedback loop: localized high current accelerates preferential Zn depletion and dendrite growth, which in turn further distorts the current field and exacerbates inhomogeneous reactions [10, 55]. The sharp increase in overpotential under high-areal-capacity cycling mainly originates from the synergistic accumulation of three factors: charge transfer overpotential induced by deteriorated electrode–electrolyte interfaces and thickened unstable solid electrolyte interphase (SEI), concentration overpotential resulting from hindered Zn^2+^ transport under lean-electrolyte conditions, and activation overpotential caused by the rapid loss of electrochemically active sites due to localized Zn exhaustion. The coupled escalation of these three kinetic components directly triggers the abrupt overpotential surge and premature battery failure.

Therefore, to achieve stable cycling performance under high-ZUR conditions, it is crucial to suppress dendrite growth and enhance the reversibility of the Zn anode. The reversibility of the Zn anode can be measured by the average CE (ACE) in electrochemical tests. The higher the ACE, the greater the reversibility of the Zn plating/stripping process. This means that the ineffective loss of Zn during cycling is minimized, increasing the likelihood of achieving stable cycling performance under the condition of a limited Zn anode. To reveal the decisive impact of ACE on the cycling performance of high-ZUR ZIBs, as Fig. 2c shows, we conducted a simple quantitative calculation under extreme conditions (ZUR = 100%). Typically, to meet commercial application requirements, the capacity retention after 1000 cycles must reach 80%. We assume that the cathode remains stable and the Zn utilization in the electrolyte is 100%. To achieve a 1000-cycle life span, the ACE of the Zn anode must exceed 99.98%. When the ZUR of the Zn anode is only 99%, the capacity of the ZIBs rapidly decays to zero with fewer than 500 cycles.

**Fig. 3:**
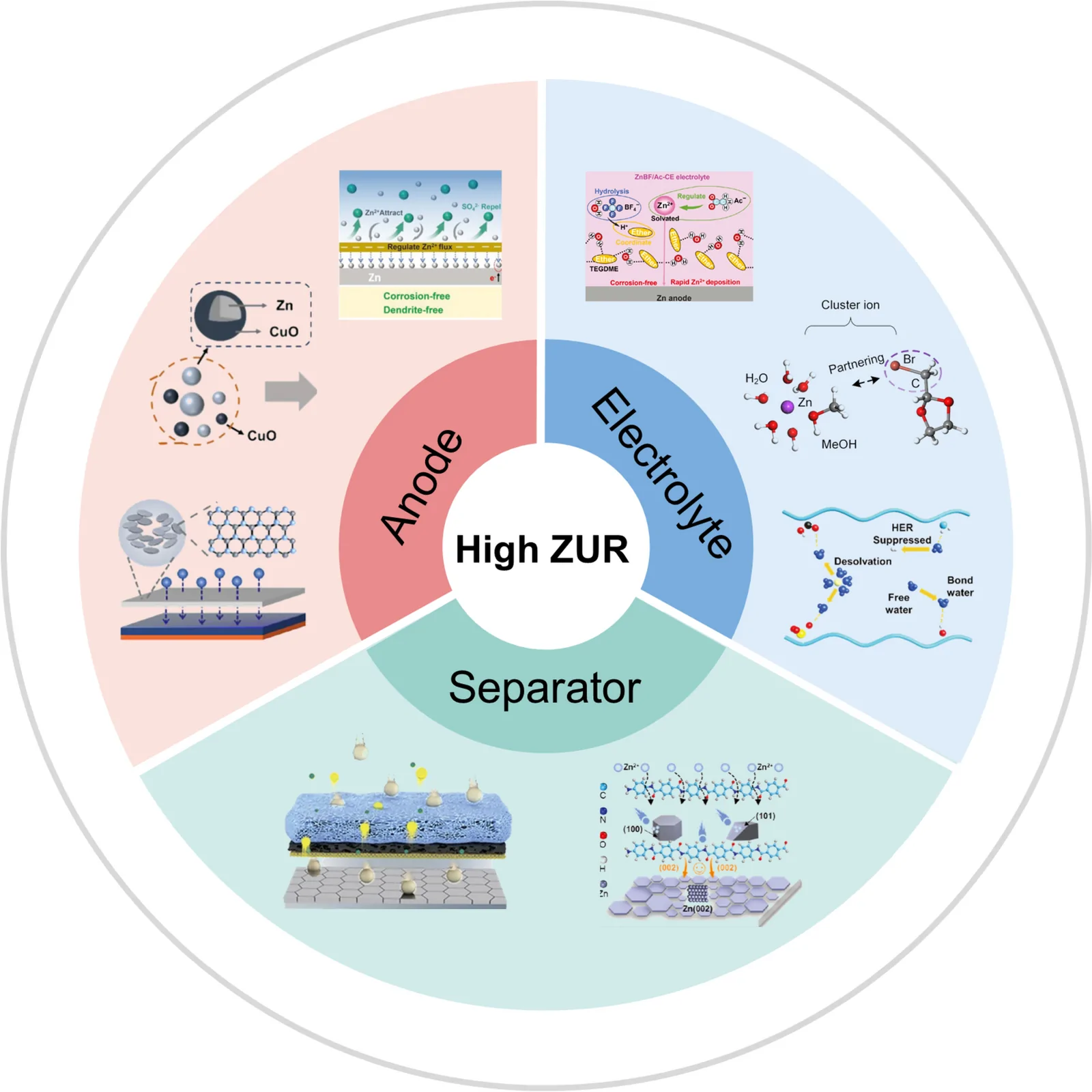
Summary of various strategies proposed to enhance the stability of Zn anodes under high-ZUR conditions

To improve the stability of the Zn anode under high-ZUR conditions, various strategies have been proposed, primarily focusing on electrode [56–58] (protective layer modification, structure design), electrolyte optimization [44, 59, 60], and separator design [61, 62]. These approaches aim to regulate the electrode–electrolyte interface to stabilize the Zn anode (Fig. 3). The main objectives are: first, to control dendrite growth and prevent uneven Zn deposition at high areal capacity, which can lead to battery short-circuit failure; second, to suppress or block a series of side reactions caused by active water molecules; and last, to mitigate concentration polarization that may occur at the electrode–electrolyte interface during the deposition/stripping process. These objectives align with the general direction of Zn anode protection strategies, but they impose more stringent requirements. In the following sections, we will summarize the work related to Zn anodes and ZIBs with high ZUR from these three perspectives. Finally, we will discuss the challenges and prospects for constructing Zn anodes and ZIBs with high ZUR, hoping to provide comprehensive guidance for advancing the commercialization of ZIBs.

## Zn Anodes

The Zn anodes used in ZIBs with high ZUR can currently be classified into three main categories: (1) *Direct use of thin Zn foil as the anode:* This method often faces issues like dendrite formation and corrosion due to the unstable interface between the anode and electrolyte [63]. Additionally, thin Zn foils are prone to pulverization and cracking during repeated Zn plating/stripping cycles, leading to the loss of active Zn and reduced battery performance and life span. (2) *Zn powder composite anode:* Zn powder offers several advantages such as low cost, large-scale processability, and adjustable structures, making it an attractive anode material. However, compared to Zn foil, Zn powder exhibits higher electrochemical activity and may encounter more severe side reactions during repeated plating/stripping processes [52]. To address these issues, many strategies have been proposed, such as structural modifications to the Zn powder, composites with other materials [64], binder modifications [65], and current collector design [66]. (3) *Pre-deposited Zn on a substrate:* This method allows for precise control over the capacity of Zn and, through the design and modification of the substrate, better regulation of the Zn plating/stripping behavior. The substrate design ensures that the Zn anode maintains stable electrical contact and structural integrity during repeated cycling, effectively enhancing high ZUR in Zn anodes [67].

For the purpose of enhancing ZUR in Zn anodes, the first category is typically addressed through “protective layer” strategies to stabilize the anode–electrolyte interface, while the second and third categories are summarized under the umbrella of “Structure design of Zn anodes.” Next, we will summarize and discuss the related works on achieving Zn anodes with high ZUR through anode modifications.

### Protective Layer

**Fig. 4:**
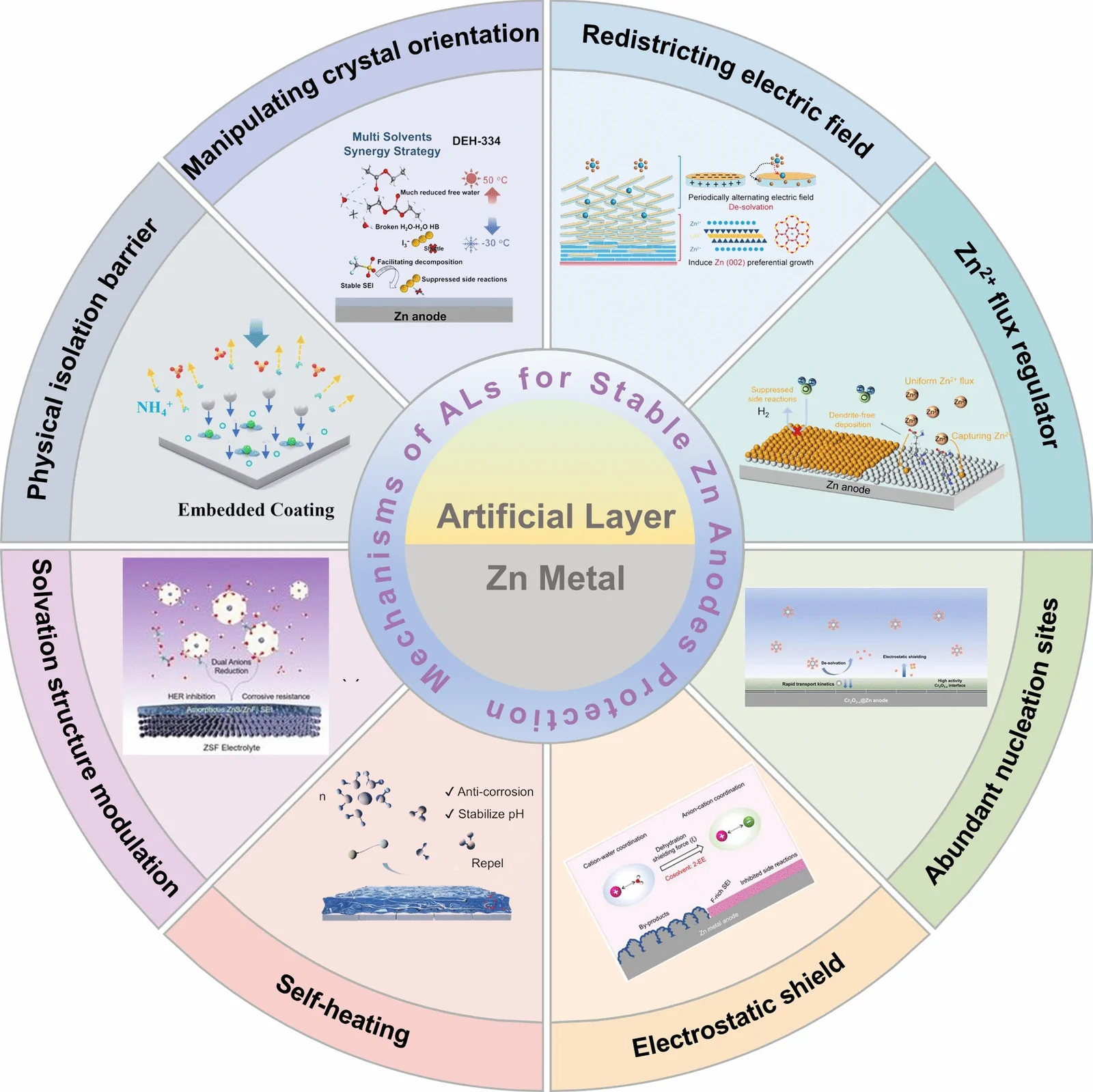
Summary of mechanisms of artificial layers for stable Zn Anodes Protection

The interface between the metal Zn anode and the electrolyte plays a crucial role in determining the stability of the Zn anode and the overall performance of the battery [68–70]. To enhance the performance of Zn anodes, suppress side reactions, and extend their cycling life while improving CE, the artificial construction of protective layers on the Zn anode has been proven to be an effective strategy. Artificial layers should not be regarded only as static protective barriers. They may also modulate the electrolyte-facing interface by altering local electric fields, H_2_O orientation, Zn^2+^ transport pathways, and the accessibility of reactive water molecules. Currently, the mechanisms behind the protective layer mainly include the following eight mechanisms [71], as shown in Fig. 4:

1. *Physical barrier:* The protective layer acts as a physical barrier that effectively isolates the Zn anode from direct contact with the electrolyte, reducing side reactions and improving the chemical stability of the Zn anode [72–74].
2. *Provision of nucleation sites:* The protective layer provides additional nucleation sites for Zn ions, promoting uniform deposition of Zn and suppressing dendrite growth, thereby enhancing the electrochemical performance of the Zn anode [75].
3. *Zn*^*2+*^* flux regulator:* By modulating the flux of Zn ions, the protective layer can effectively control the rate of Zn^2+^ ion transfer and deposition, achieving a more uniform and stable deposition/dissolution process [76].
4. *Redistribution of electric field:* The protective layer creates a redistributed electric field on the surface of the Zn anode, which helps to uniformly distribute the current density and alleviate the formation of Zn dendrite [69].
5. *Electrostatic shielding:* The protective layer can shield the interaction between charged particles on the Zn anode surface and those in the electrolyte through electrostatic effects, thus reducing the driving force for dendrite growth and suppressing side reactions on the anode [77].
6. *Crystal orientation control:* The protective layer can alter the deposition behavior of Zn^2+^ ions and induce preferential deposition on specific crystal facets, optimizing the crystal orientation of the Zn deposits, reducing irregular deposition, and suppressing dendrite growth [35].
7. *Solvation structure modulation:* By adjusting the solvation structure of Zn^2+^ ions in the electrolyte, the protective layer optimizes the deposition/dissolution behavior of Zn^2+^ ions on the Zn surface, thereby improving the electrochemical stability and cycling performance of the Zn anode [39, 78].
8. *Self-healing function:* Certain protective layer materials possess self-healing properties, meaning that if local damage occurs on the surface of the Zn anode, the protective layer can automatically repair itself, restoring its protective function and ensuring the long-term stability of the Zn anode [79].

In summary, strategies based on protective layers provide strong support for regulating the uniform and stable deposition/dissolution of Zn to achieve Zn anodes with high ZUR. In the next section, we will summarize and analyze the current research on Zn anodes with high ZUR based on protective layer strategies.

**Fig. 5:**
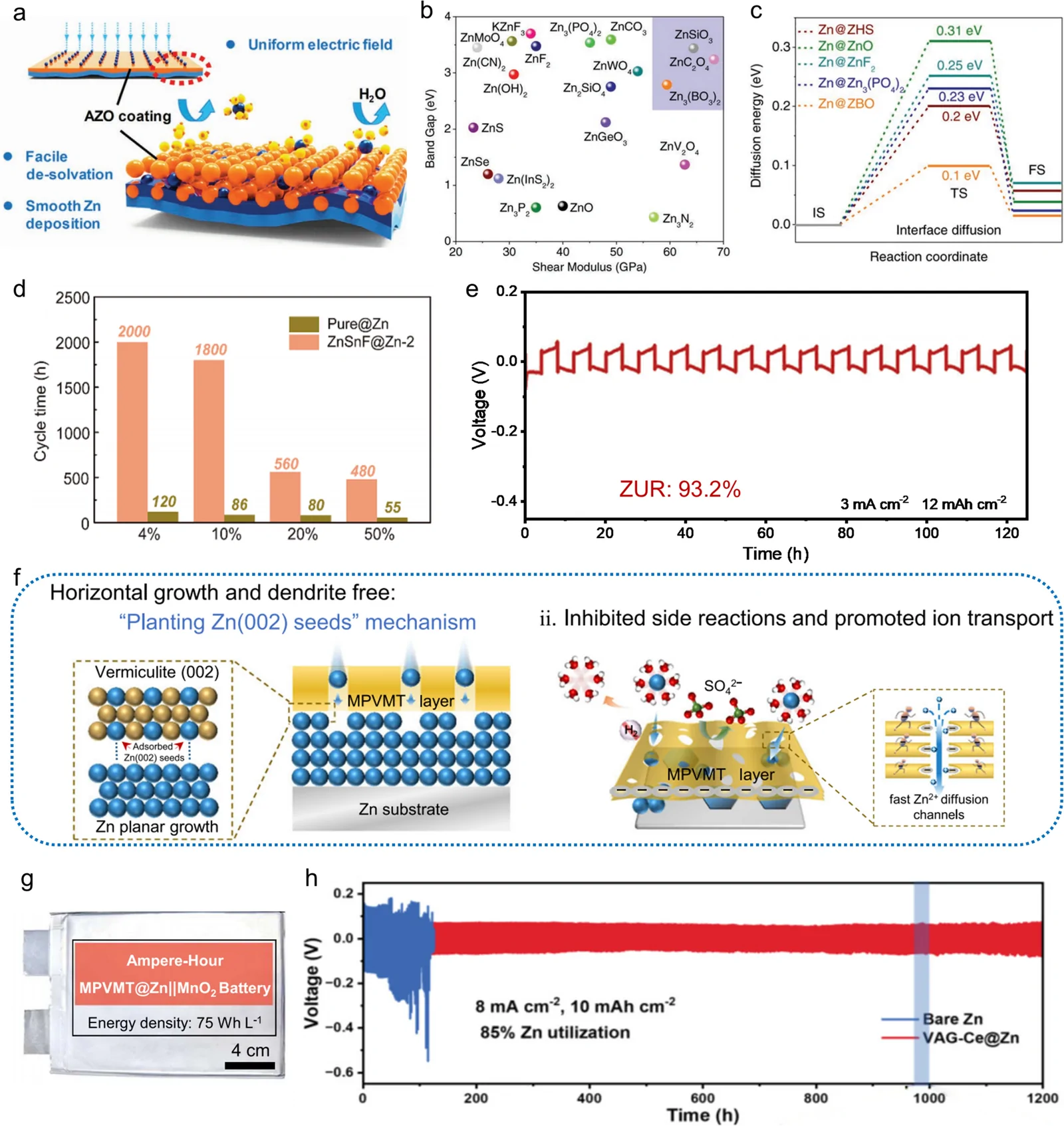
**a** Schematic illustration of Zn deposition process on Zn@AZO. From Ref. [80]. Copyright 2021, Wiley–VCH. **b** Bandgaps and shear moduli of potential SEI candidates. **c** Zn diffusion energy at the interfaces of Zn@ZHS, Zn@ZnO, Zn@ZnF_2_, Zn@Zn_3_(PO_4_)_2_, and Zn@ZBO. From Ref. [81]. Copyright 2023, Wiley–VCH. **d** Cycling performances of symmetric Zn||Zn batteries with Pure@Zn and ZnSnF@Zn-2 under various Zn utilizations. From Ref. [82]. Copyright 2024, American Chemical Society. **e** Performances of a symmetrical Zn battery with LPL under 3 mA cm^−2^, 12 mAh cm^−2^. From Ref. [83]. Copyright 2024, Wiley–VCH. **f** Schematic of MPVMT layers based on extended substrate screening strategy (ESSS) to stabilize Zn anodes. **g** Optical image of an Ah-level MPVMT@Zn||MnO_2_ pouch cell. From Ref. [84]. Copyright 2024, Springer Nature. **h** Cycling performance of the symmetric cell at 8 mA cm^−2^, 10 mA h cm^−2^ under 85% ZUR. From Ref. [57]. Copyright 2023, Wiley–VCH

To improve the durability of the Zn anode at high ZUR, Huang et al. [80] proposed constructing an aluminum-doped zinc oxide (AZO) protective layer on the Zn anode surface. The AZO layer inhibits side reactions by isolating the active Zn from the bulk electrolyte and accelerates the desolvation of hydrated Zn^2+^ ions, leading to uniform Zn deposition dynamics via evenly distributed electric fields (Fig. 5a). Based on this, Zn@AZO symmetric cells maintained stable cycling performance over 200 h at a ZUR of 80%. Yang et al. [81] theoretically proposed a method for screening potential artificial SEI protective layers on Zn anodes from the perspectives of dendrite suppression ability and charge transfer characteristics. First, potential materials were screened based on basic properties, shear modulus, and band gap. Then, based on the energy barrier for Zn^2+^ diffusion, the charge transfer properties were further calculated, and the product of interface energy (*γ*) and Young’s modulus (E) was used to elucidate the dendrite suppression capability (Fig. 5b, c). Theoretical and experimental results indicated that Zn_3_(BO_3_)_2_ (ZBO) effectively promoted uniform Zn deposition and lateral growth while suppressing side reactions. The Zn@ZBO symmetric cell, with a capacity of 10 mAh cm^−2^ (ZUR of 60%), exhibited a long cycle life of over 250 h. Additionally, the Zn@ZBO||MnO_2_ full cell showed improved cycling performance under limited electrolyte (10 µL mAh^−1^), limited Zn supply (*N*/*P* ratio = 2.3), and high area capacity (5 mAh cm^−2^). Huang and Luo et al. [82] constructed an ion-sieving protective layer (ZnSnF@Zn) on a thin Zn anode using a chemical substitution strategy. The ion sieve facilitates the transport and desolvation of Zn^2+^ ions at the anode/electrolyte interface, reducing the Zn deposition overpotential and suppressing side reactions. As shown in Fig. 5d, the stability of symmetric cells based on Pure@Zn is poor when ZUR ranges from 4 to 50%. In contrast, ZnSnF@Zn-2 achieves 480 h of stable cycling at 50% ZUR. Wu and Jiao developed a simple and effective self-adsorption strategy to fabricate a lysozyme protective layer (LPL) on the Zn metal surface [83]. The hydrophobic nature and conformational changes of LPL can expel free water and modify the electric double layer (EDL) on the Zn anode, effectively suppressing side reactions. Thus, at a high ZUR of 93.2%, symmetric Zn cells can maintain stability for over 220 h in Fig. 5e. The LPL also enables the Zn/Zn_0.25_V_2_O_5_ pouch cell to operate stably under a low *N*/*P* ratio of 2.1.

Zhou et al. developed a large-scale, green method to prepare a two-dimensional hydrophilic insulating monolayer of porous vermiculite (MPVMT) as a protective coating for the Zn anode [84]. Due to MPVMT’s low lattice mismatch (*δ* = 0.38%), the “2dZn (002)” structural units, and strong adsorption of Zn deposits, the coating induces Zn (002) orientation growth via a “seeded Zn (002)” mechanism. Additionally, the hydrophilic functional groups and negative charge on the surface of MPVMTs greatly promote the desolvation of Zn^2+^, homogenizing the electric field and suppressing side reactions in synergy (Fig. 5f). Moreover, under harsh conditions (51% ZUR and low *N*/*P* ratio of 1.9), the MPVMT coating enables the Zn||I_2_ battery to maintain nearly 100% capacity after 200 cycles. The assembled MPVMT@Zn|MnO_2_ pouch cell achieves a capacity of 1.25 Ah and an energy density of 75 Wh L^−1^ (Fig. 5g).

In addition to using inorganic coatings, CeO_2_ aerogel (VAG-Ce) interface layers have also been reported to integrate the advantages of Zn^2+^ selectivity, porosity, and lightweight oxygen vacancies, thus enhancing the stability of Zn anodes under high ZUR [57]. The abundant oxygen vacancies exposed on the VAG-Ce surface can strongly capture SO_4_^2−^ ions, forming a negatively charged layer that attracts Zn^2+^ ions and accelerates their migration dynamics. This layer also repels additional anions, effectively suppressing the by-products formation. As a result, the VAG-Ce@Zn anode can achieve a high cycling life span of 1200 h at 85% ZUR, as shown in Fig. 5h.

### Structure Design of Zn Anodes

#### Zn Powder-Based Anode

Zn powder is also widely used for large-scale production of Zn anodes with controllable Zn capacity and high ZUR. The common preparation method involves mixing Zn powder with a conductive agent and binder, followed by casting it onto an inert substrate (such as Cu foil) [85]. This preparation method is compatible with existing electrode technologies and facilitates large-scale manufacturing. However, the high specific surface area of Zn powder exposes it to higher reactivity, leading to issues such as HER, corrosion, uncontrolled dendrite growth, and poor mechanical strength [86]. Additionally, when Zn powder is used as the anode, selecting an appropriate current collector is often necessary, and there are some extra challenges compared to using conventional Zn foils [66]: (1) A micro-galvanic cell may form between the Zn powder and the Cu foil current collector, causing corrosion; (2) The deposition/stripping of Zn powder on the current collector results in volume expansion/contraction of the electrode, which further leads to the separation of active materials from the electrode; (3) Smaller Zn powder particles dissolve preferentially, and deposition occurs around larger particles, generating edge effects and forming dendrites, which eventually puncture the separator and cause battery failure. Previous studies have shown that through rational optimization design, including coating protective layers, designing conductive scaffolds, and improving binders, the stability and high ZUR of Zn powder-based anode can be effectively enhanced, offering promise for the further commercialization of aqueous ZIBs.

**Fig. 6:**
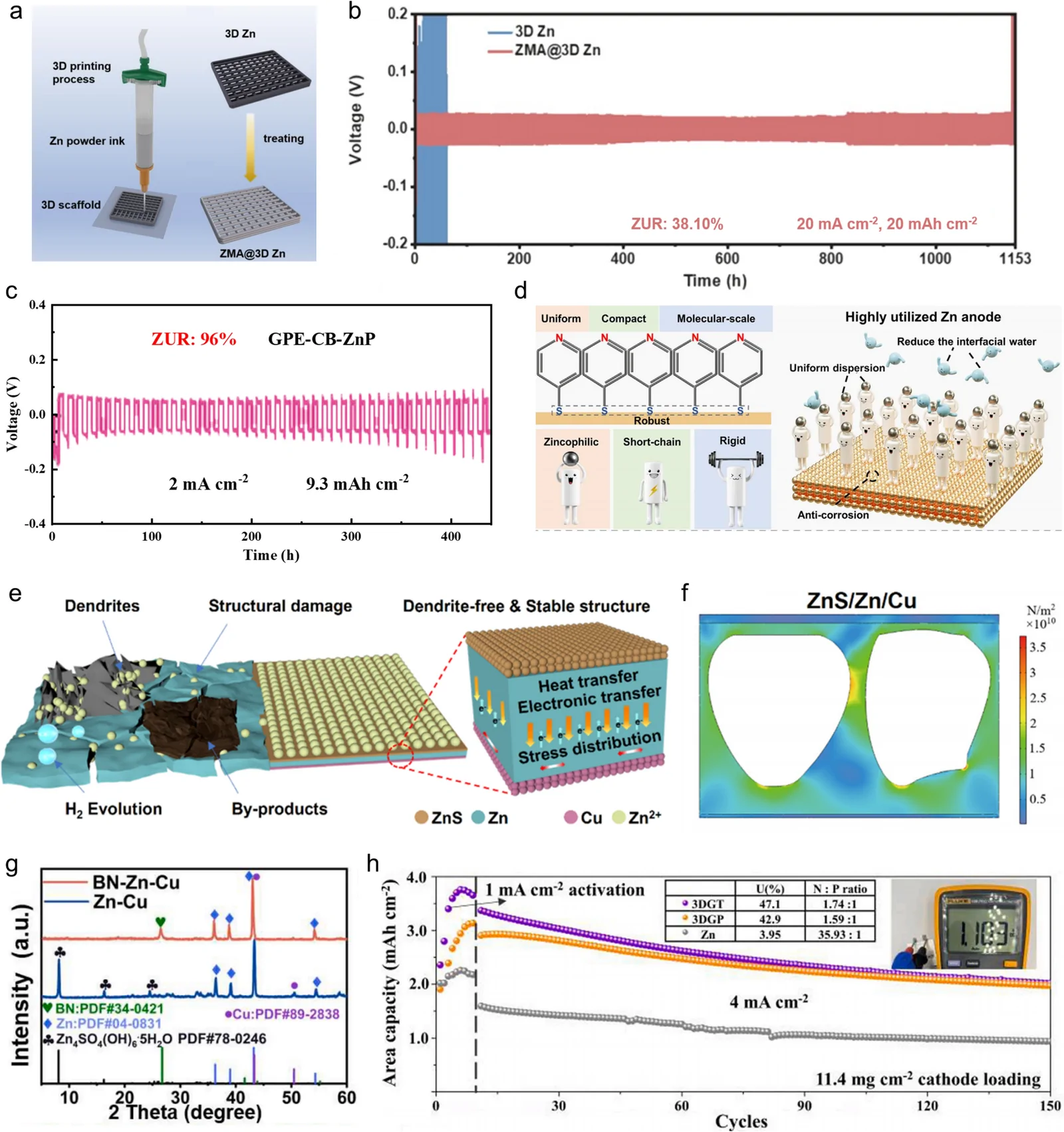
**a** Schematic illustration of the preparation process of 3D Zn scaffold. **b** Voltage profiles of symmetric cells with bare 3D Zn and ZMA@3D Zn electrodes at current density/capacity of 20 mA cm^−2^/20 mA h cm^−2^. From Ref. [87]. Copyright 2025, Science China Press. **c** Cycling stability of Zn||Zn symmetric cells configured with GPE-CB-ZnP, tested at 2 mA cm^−2^ and 9.3 mAh cm^−2^. From Ref. [88]. Copyright 2025, Wiley–VCH. **d** Schematic illustration of the 4Mpy SAMs regulating the Zn deposition and suppressing the interfacial side reactions. From Ref. [89]. Copyright 2025, Elsevier. **e** Schematic diagram of the mechanism of double-sided engineering. **f** Stress distribution of the ZnS/Zn/Cu electrode obtained through finite element simulation. From Ref. [90]. Copyright 2024, Royal Society of Chemistry. **g** XRD patterns of Zn–Cu and BN–Zn–Cu anodes after cycling. From Ref. [56]. Copyright 2024, Elsevier. **h** Cycling performance of 3DGT@Zn//V_2_O_5_, 3DGP@Zn//V_2_O_5_, and Zn//V_2_O_5_ in coin cell at 4 A g^−1^. From Ref. [91]. Copyright 2023, Elsevier

Wu and Lin designed a high ZUR, non-dendritic Zn anode through the spontaneous reaction between commercial Zn powder and graphene oxide (Zn–G), without the need for any binders [86]. The functional groups remaining on the graphene sheets provide the electrode with integrity and good mechanical properties. Furthermore, the wrapped graphene sheets reduce the side reactions of Zn particles by minimizing the contact between Zn and water molecules. As a result, in a Zn–G||MnO_2_ full cell, a capacity retention of 74.5% was achieved after 1000 cycles at a low *N*/*P* ratio of 3. As shown in Fig. 6a, Guan et al. [87] fabricated a multifunctional carbonyl-containing zinc methacrylate (ZMA) layer on the surface of a three-dimensional Zn powder anode (ZMA@3D Zn) through in situ modification. On one hand, the highly electronegative and nucleophilic carbonyl groups in the ZMA layer facilitate the desolvation process of Zn^2+^ ions, which aids in Zn^2+^ transport and uniformity. On the other hand, the hydrophobic carbon chains in the ZMA act as a protective layer, reducing the direct contact between Zn powder and free water, thereby suppressing side reactions at the zinc anode. Based on this, the ZMA@3D Zn anode demonstrated stable cycling for over 1100 h at a ZUR of 38.1% and a capacity of 20 mAh cm^−2^ (Fig. 6b).

#### Pre-Deposited Zn Anodes

In addition to using thin Zn foils and composite Zn powder anodes, pre-depositing Zn onto modified substrates as the anode is also an effective strategy to enhance the electrochemical performance of Zn anodes with high ZUR [92]. As mentioned earlier, uneven Zn plating/stripping can lead to the complete depletion of local Zn metal, eventually causing the fracture of the Zn foil. This fracture disrupts both the Zn source and electronic conduction in the anode, accelerating battery degradation [67]. In contrast to thin Zn anode designs, pre-deposited Zn onto inert metal or nonmetal substrates (such as Ti and Cu) as the anode ensures good electrical contact between the Zn and the substrate, thereby prolonging the cycling life of the Zn anode at high ZUR [93]. Moreover, Zn electroplating technology has been well established in traditional electroplating industries, making large-scale production of electroplated Zn anodes feasible.

Chen et al. [63] developed an ultra-thin zinc composite anode (24 μm) with a protective hydrophobic layer of covalent (C_2_F_4_)_n_ chains and fluorinated carbonized components ((C_2_F_4_)_n_–C@Cu) as the substrate for Zn deposition on Cu foil. This layer exhibits excellent hydrophobicity, which helps prevent side reactions of Zn caused by damage from active water molecules. In combination with the semi-ionic fluorine species as zincophilic sites, the matrix guides uniform and dense Zn deposition, resulting in the fabrication of the ultra-thin Zn anode. A symmetric cell based on (C_2_F_4_)_n_–C@Cu@Zn showed stable cycling for 900 h at a ZUR of 40%. Zhou et al. [88] developed a comprehensive electrochemical–mechanical regulation strategy to construct a viscoelastic Zn powder anode (GPE-CB-ZnP) by integrating branched oxygen-rich oligomers (GPE) and spherical carbon black fillers (CB). The weak zinc coordination effect and expansive free volume of GPE facilitate rapid Zn^2+^ flux, while its ether–oxygen moieties immobilize water to suppress parasitic reactions, and the spherical CB mitigates stress concentration. Thus, at an ultra-high ZUR of 96%, symmetric Zn cells can maintain stable cycling for over 430 h in Fig. 6c. The GPE-CB-ZnP anode also enables Zn-based full cells to operate stably under low *N*/*P* ratios (1.78–4.89) with high capacity retention. Zhu et al. [89] designed a zincophilic short-chain aromatic molecule, 4-mercaptopyridine (4Mpy), to construct a self-assembled monolayer film (SAMs) on Cu substrates, achieving a high-ZUR Zn anode. As shown in Fig. 6d, 4Mpy can firmly attach to the Cu substrate via Cu–S bonds, forming a dense and uniform monolayer film that effectively isolates the anode surface from water, thus eliminating side reactions associated with water molecules. As a result, the Zn/4Mpy/Cu electrode enables the symmetric cell to stably cycle for over 180 h at a high ZUR of 90%. Additionally, a 4Mpy/Cu||graphite cell without an anode demonstrated 150 cycles with no significant capacity degradation. Luo and Mai designed an ion tunnel matrix substrate composed of carbon cloth decorated with cubic Prussian blue analogs (PBA@CC) [94]. The well-spaced PBA nanocubes act as Zn^2+^ ion tunnels, facilitating highly reversible and dendrite-free plating/stripping to achieve high ZUR. A Zn-PVO pouch cell with a pre-deposited Zn anode based on the PBA@CC substrate exhibited a ZUR as high as 83% (*N*/*P* = 1.2). The pouch cell with high ZUR also demonstrated lightweight properties, achieving an initial energy density of up to 249 Wh kg^−1^ based on the anode and cathode.

Generally, most substrates exhibit a top-layer Zn deposition mode, where side reactions between the deposited active Zn and the electrolyte result in suboptimal ZUR [56]. To address the issue of traditional top-layer Zn deposition on substrates, dual-sided engineered anodes based on a sandwich structure have emerged as an effective strategy to enhance the stability of Zn anodes under high ZUR. Guan proposed a dual-sided engineering strategy (ZnS/Zn/Cu) for stabilizing Zn anodes [90]. In this design, the top ZnS layer suppresses corrosion and hydrogen evolution while promoting Zn^2+^ flux, whereas the bottom Cu layer stabilizes the electronic conduction pathway, reduces stress concentration, and accelerates localized heat transfer (Fig. 6e, f). This dual-sided engineering synergistically induces spatially confined reversible Zn deposition behavior, effectively improving the plating/stripping reversibility at high ZUR. Specifically, the ZnS/Zn/Cu anode demonstrated stable cycling for 300 h at a ZUR of 85.5%. The application of a boron nitride (BN) layer on Cu foil as a substrate to achieve a sandwich-structured anode (BN–Zn–Cu) has also been proven to enhance the ZUR [56]. The insulating BN layer acts as a protective barrier for Zn deposition on the top of the anode while facilitating the transport of Zn^2+^ ions. The zincophilic Cu substrate layer, as the bottom layer of the anode, promotes zinc deposition without dendrite formation. Under the synergistic effect of the BN layer and substrate, this sandwich-structured anode significantly suppresses side reactions during cycling, exhibiting excellent reversibility (Fig. 6g). The BN–Zn–Cu symmetric cell achieved a ZUR of 66.7% at an areal capacity of 4 mAh cm^−2^.

For the commonly used two-dimensional Zn anodes (Zn foil) in ZIBs, the formation of Zn dendrites at the anode/separator interface often leads to a negative correlation between electrochemical performance and ZUR. To overcome the limitations of the two-dimensional geometric design of Zn anodes, Zeng and Zhao fabricated 3D-printed graphene arrays (3DGs) to simultaneously improve the reversibility and utilization rate of Zn anodes [91]. The highly ordered 3D array structure not only accommodates the volume changes during the reversible zinc deposition/dissolution process but also buffers the interaction between metallic Zn and the separator, thus preventing short-circuiting. As a result, pouch cells using 3DGs@Zn anodes and V_2_O_5_ cathodes can achieve a stable lifetime at a low *N*/*P* ratio of 1.74 (Fig. 6h).

**Table 1:** Summary and comparison of electrochemical performance for representative Zn anodes with high utilization rates (ZURs), including various modification strategies across Zn foil, Zn powder, and pre-deposited Zn under practical testing conditions

Category	Modification strategy	Areal metrics (mA cm^−2^, mAh cm^−2^)	ZUR (%)	Cycling life (h)	References
Zn foil	Zn–Sn (200)	5, 4	80	900	[48]
	AZ-250	4.7,4.7	80	250	[95]
	AZH@Zn	4, 40	68.32	120	[96]
		5,50	85.4	120	
	Zn-PG	5, 10.2	90%	1000	[97]
	ILG-Zn	0.5, 1.8	90%	400	[98]
	LPL	1, 10	77.7	1200	[83]
		3, 12	93.2	120	
Zn powder	pZn/In	10, 2.5	38.5	280	[99]
	Zn-ME	6, 4	60	120	[100]
	ZMA@3D Zn	20, 20 30,30	38.1 57.16	1153 220	[87]
	GPE-CB-ZnP	1, 1	30	600	[88]
		2, 9.3	96	400	
	Calendared SnO_2_@Zn	5.52	40	300	[101]
	ZnP-β-CD	10, 5	80	200	[102]
	PL-ZnP	10, 5	75	500	[64]
Pre-deposited Zn	(C_2_F_4_)n–C@Cu	5, 2	40	900	[63]
	ZnFe-PBA@CC	5, 0.5	100	1320	[94]
	Zn (002)	20, 10	37.5	170	[93]
	ZnS/Zn/Cu	5, 5	85.5	300	[90]
	4Mpy/Cu	5, 5 10, 9	33.3 90	1000 200	[89]
	BN–Zn–Cu	4, 4	66.7	180	[56]

Overall, the development of high-Zn-utilization anodes requires a transition from “protecting excess Zn” to “efficiently managing limited Zn”. To provide a more direct and quantitative overview of recent progress from the anode perspective, representative studies on high-ZUR Zn anodes are summarized in Table 1 according to Zn foil protection, Zn powder anodes, and pre-deposited Zn anodes. The table lists the reported Zn utilization rate, areal capacity, current density, cycling performance and Coulombic efficiency.

## Electrolytes

**Fig. 7:**
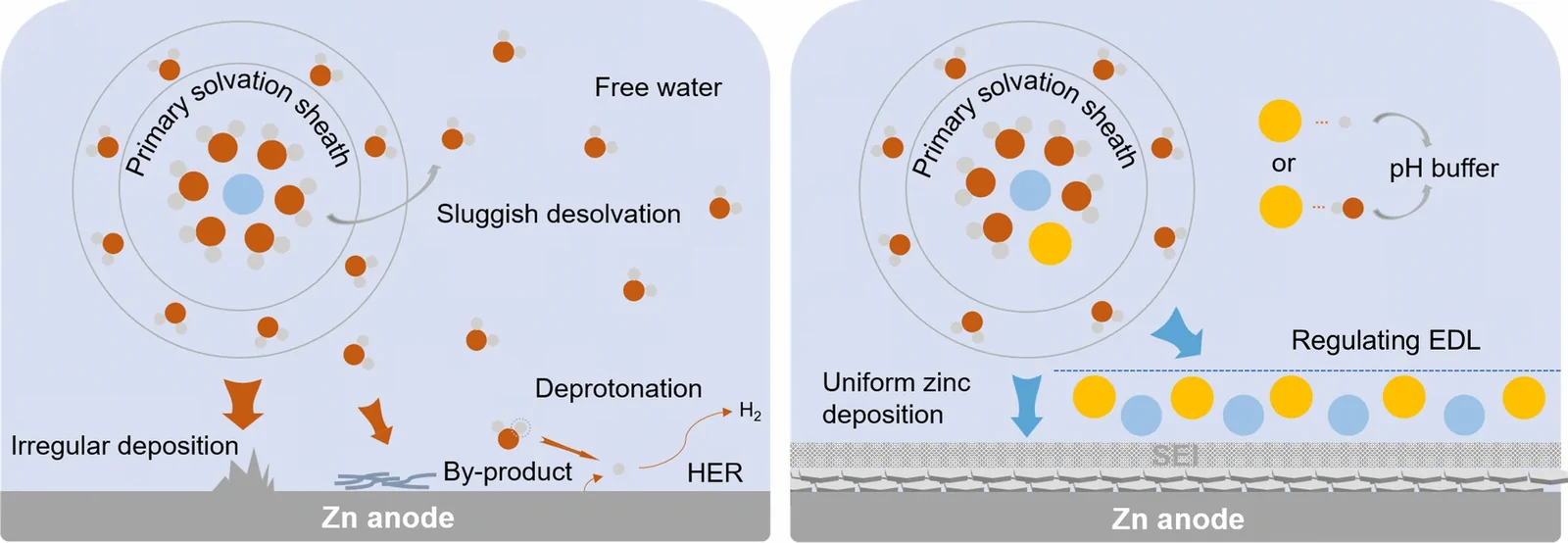
Schematic diagram illustrating the mechanism of electrolyte additives

As mentioned above, a series of side reactions caused by active water molecules in the electrolyte directly affect the performance of the Zn anode, making it difficult to improve the ZUR. Appropriate electrolyte regulation cannot only change bulk water activity, but also reconstruct the dynamic interfacial H_2_O configuration, including Zn^2+^ hydration shells, hydrogen-bonding networks, and the orientation of water molecules under polarization. Therefore, electrolyte regulation is crucial for enhancing the utilization, life span, and CE of the Zn anode [103]. Currently, the main strategies for electrolyte regulation can be divided into two categories: the introduction of electrolyte additives and the fabrication of gel electrolytes. The principles of these two methods are described as follows:

The electrolyte additives strategy involves adding specific chemicals to the electrolyte to regulate the electrochemical behavior of the Zn anode and improve battery performance [104, 105]. The mechanisms of action of additives typically include: altering the solvation structure of Zn^2+^ ions, forming a SEI layer on the Zn anode surface, modulating electrostatic repulsion caused by the electric double layer (EDL) of counterions, inducing selective crystal plane deposition of Zn, and serving as a pH buffer. These actions suppress dendrite growth, reduce hydrogen evolution reactions, and mitigate corrosion phenomena, thereby improving the utilization of the Zn anode (Fig. 7). Gel electrolytes are a type of semisolid electrolyte material formed by combining a polymer matrix with electrolyte solvents. Gel electrolytes effectively restrict water molecules within the matrix through hydrogen bonding, thus inhibiting the activity of water molecules and reducing side reactions caused by active water molecules. This prevents issues such as electrolyte leakage and short-circuiting.

In the next sections, we summarize a series of representative studies to improve ZUR through electrolyte engineering strategies.

### Additives

**Fig. 8:**
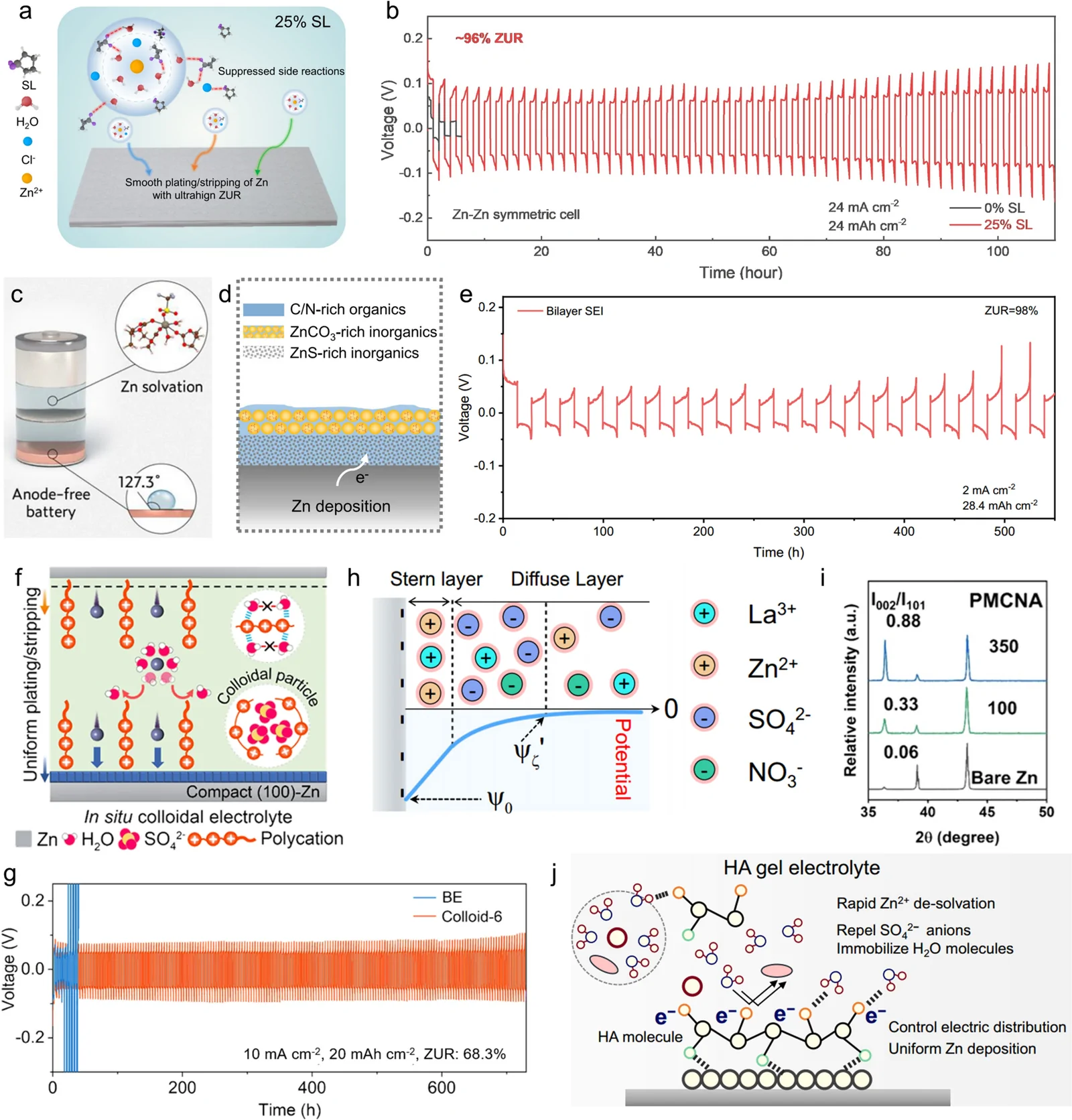
**a** Schematic illustration of the solvation structures of Zn ions in the electrolytes with 25% SL and their interfacial reactions at the electrode/electrolyte. **b** Performance of the Zn–Zn cell in the electrolytes with 0% SL and 25% SL at 24 mA cm^−2^ and 24 mA h cm^−2^ with 96% ZUR. From Ref. [55]. Copyright 2023, Wiley–VCH. **c** Schematic illustration of the Zn-ion solvation structure, and the resultant hydrophobic interphase. From Ref. [115]. Copyright 2022, American Chemical Society. **d** Schematic presentation of the bilayer SEI. **e** Long-term stability of symmetric cells at 28.4 mAh cm^−2^ and 2 mA cm^−2^ (ZUR = 98%). From Ref. [117]. Copyright 2024, Springer Nature. **f** Schematic diagram of Zn deposition/stripping behavior in in situ colloidal electrolyte. **g** Cycle performance of Zn–Zn symmetric cell at 10 mA cm^−2^ and 20 mAh cm^−2^. From Ref. [118]. Copyright 2026, American Chemical Society. **h** Electric double layer of the Zn deposits in La^3+^-ZS (ZnSO_4_) electrolytes. From Ref. [119]. Copyright 2022, Springer Nature. **i** XRD patterns of Zn metal with PMCNA after 100 and 350 cycles. From Ref. [120]. Copyright 2023, Wiley–VCH. **j** Schematic illustration of the regulation mechanism of HA gel electrolyte on Zn electroplating. From Ref. [121]. Copyright 2023, Springer Nature

In the solvation structure of Zn^2+^ (Zn(H_2_O)_6_^2+^), the –OH bond of H_2_O is weakened, which promotes the competitive hydrogen evolution reaction during the Zn plating process, leading to low plating/stripping CE [106–110]. Adding organic solvents with functional groups to aqueous electrolytes is an inexpensive and effective strategy to reduce the activity of H_2_O. These solvents can easily form hydrogen bonds with H_2_O molecules, disrupting the original H_2_O hydrogen-bonding network [111, 112]. On the other hand, organic solvents typically exhibit stronger interactions with Zn^2+^, allowing them to participate in the solvation shell of Zn^2+^ [113, 114]. Chen et al. [55] introduced sulfolane (SL) into the electrolyte, which reshaped the primary solvation shell of Zn^2+^, significantly reducing the side reactions at the Zn anode and achieving a high ZUR under high areal capacity (Fig. 8a). As shown in Fig. 8b, in an electrolyte containing 25% SL, symmetric Zn–Zn and asymmetric Cu–Zn cells can achieve up to 96% ZUR (24 mAh cm^−2^). Furthermore, at 67% ZUR, the Zn–V_2_O_5_ full cell can stably cycle for 500 cycles with an energy density of 180 Wh kg^−1^. Alshareef et al. [115] designed a hybrid electrolyte based on the salting-out effect, consisting of a mixture of propylene carbonate and water, as a proof of concept (Fig. 8c). This hybrid electrolyte effectively modulates the solvation structure of Zn^2+^, providing a well-formed Zn^2+^ solvation shell, which ensures the stable operation of anode-free Cu–ZnMn_2_O_4_ batteries. Hu et al. [44] developed a dual-salt/dual-solvent electrolyte composed of Zn(BF_4_)_2_/Zn(Ac)_2_ in a water/TEGDME (tetraethylene glycol dimethyl ether) solvent system. This unique dual-salt/dual-solvent system exhibits a synergistic effect of “internal co-salts and external co-solvents.” Based on this electrolyte, the Zn anode demonstrates high reversibility at a high utilization rate of 60%. Sun et al. [116] demonstrated that introducing 0.1% phosphoramidite (PA) molecules can act as an effective interface modifier. The solvation structure of Zn^2+^ transitions from being primarily water-based in the bulk electrolyte to being dominated by PA, with anions participating in interactions at the Zn surface. Symmetric Zn–Zn cells with high ZUR (52.2% at 50 mAh cm^−2^ and 92.4% at 88.5 mAh cm^−2^) achieve lifetimes of 392 and 140 h, respectively.

In addition to altering the solvation structure of Zn^2+^ ions, in situ construction of a SEI on the Zn anode through electrolyte additives is also an effective strategy to stabilize the Zn anode/electrolyte interface and improve the ZUR [122, 123]. The SEI not only isolates the Zn anode from direct contact with active water molecules, thereby preventing side reactions like HER and chemical corrosion, but it also ensures uniform Zn^2+^ transport, promoting even Zn plating/stripping and significantly improving CE [124]. Chen et al. [117] proposed the addition of 10 mmol L^−1^ 1,3-dimethyl-2-imidazolidinone (DMI) to build a robust bilayer SEI. The bilayer SEI consists of a crystalline outer layer rich in ZnCO_3_ and an amorphous inner layer rich in ZnS. The ordered outer layer enhances the mechanical stability during cycling, while the amorphous inner layer promotes uniform Zn^2+^ transport (Fig. 8d). Consequently, the bilayer SEI enables 4800 reversible plating/stripping cycles with a CE of 99.95%. The Zn||Zn symmetric cell demonstrates an impressive lifetime of over 550 h at an area capacity of 28.4 mAh cm^−2^, with a record-high ZUR of up to 98% (Fig. 8e). Wang et al. [118] developed an in situ colloidal electrolyte via SO_4_^2−^ polycation electrostatic interaction for aqueous Zn metal batteries. Figure 8f shows that the colloidal electrolyte enables compact (100)-plane-oriented Zn deposition and uniform stripping by confining SO_4_^2−^ diffusion and disrupting water’s hydrogen-bonding network. Consequently, Zn||Zn symmetric cells in the optimized Colloid-6 electrolyte maintain stable cycling for over 700 h at 20 mAh cm^−2^ (ZUR: 68.3%) (Fig. 8g), and achieve ultra-long deep-cycling durability over 300 h even at 25 mAh cm^−2^ (ZUR: 85.4%).

Based on the Derjaguin–Landau–Verwey–Overbeek theory, the interactions between Zn deposits in aqueous electrolytes are primarily governed by Van der Waals attraction and electrostatic repulsion caused by the counterion double layer [125]. In ZnSO_4_ electrolytes, Zn tends to deposit in a dispersed and loose flaky form, indicating that the Zn deposition process is controlled by repulsive forces. Therefore, by adding electrolyte additives, the competitive adsorption of different ions on the Zn anode surface can be modified, thereby adjusting the surface charge distribution on the zinc anode, which promotes uniform Zn deposition and improves ZUR. Based on this concept, Huang and Qie proposed that weakening the double-layer repulsion force is beneficial for dense Zn deposition and regulating the charge distribution at the Zn anode/electrolyte interface (Fig. 8h) [119]. As a result, under a current density of 10 mA cm^−2^ and a ZUR of 80%, the Zn–Zn cell exhibits stable plating/stripping for 160 h. In addition, the enhanced adsorption of the amino acids glycine (Gly) and cordycepin (Cor) on the surface of the Zn anode has also been reported to significantly increase the ZUR.

In addition to the changes in the electric double layer (EDL) caused by enhanced adsorption interactions, designing additives that can be adsorbed to induce specific crystal plane orientation of Zn anode deposition has become one of the effective strategies to improve the ZUR [126]. Specifically, certain additives can interact with the surface of the Zn anode to regulate the nucleation and deposition process of Zn, thereby improving the cycling stability and efficiency of the Zn anode [48]. Currently, polymer additives have been reported to adsorb onto the surface of the Zn anode, which can, on one hand, regulate the charge distribution of the anode and optimize the EDL structure [125]; on the other hand, provide specific adsorption sites on the Zn anode surface to promote preferential deposition of Zn^2+^ ions on specific crystal planes. Therefore, it can suppress dendrite growth and improve the deposition morphology of the Zn anode. This crystal plane-oriented deposition can effectively reduce Zn dendrite formation and enhance the stability and cycle life of the Zn anode. Feng et al. [120] employed polymer additives (PMCNA) designed by copolymerizing N-acryloyl glycinamide (NAGA) and 2-methacryloyloxyethyl phosphorylcholine (MPC), to guide Zn nucleation and deposition along the (002) plane to further suppress side reactions and dendrite growth (Fig. 8i). As a result, the Zn–Zn cell demonstrated a durable lifetime of more than 420 h with ZUR 90.0%. In addition to polymers, some organic and inorganic salts have also been shown to induce preferential crystal plane deposition of Zn, thereby achieving high ZUR. Wang and Yang designed a novel electrolyte additive, 3-mercapto-1-propane sulfonic acid sodium (MPS), where the MPS anion can form an adsorption layer on the anode surface. This adsorption layer can facilitate the reduction of energy barriers associated with zinc deposition, leading to preferential deposition of Zn along the (002) direction. Based on this optimized electrolyte, Zn–Zn symmetric cells with a high ZUR of 50% demonstrated more than 800 h of stable cycling [126]. Additionally, Huang and Zhang achieved a 99% relative texture coefficient of Zn (002) deposition in the electrolyte of ZnSO_4_ + H_2_SO_4_ + KMnO_4_, which significantly improved the stability of the Zn anode under high ZUR.

It is important to emphasize that the mechanism of action of an additive is not singular; it may influence the deposition behavior of the Zn anode through multiple pathways. Each additive may optimize the performance of the Zn anode at various levels through synergistic effects, although certain optimization mechanisms may dominate under specific conditions [127]. For example, potassium hydrogen phthalate (KHP) has been proven to be a multifunctional additive for ultra-stable Zn-I_2_ full cells. On one hand, K^+^ ions act as electrostatic shielding cations, contributing to the formation of a smooth deposition morphology. On the other hand, HP^−^ can enter the first solvation shell of Zn^2+^, reducing the reactivity of H_2_O, and it remains in the primary solvation shell, ultimately contributing to the formation of the SEI, thereby accelerating charge transfer kinetics. Additionally, in situ pH monitoring near the Zn surface demonstrated that KHP additives function as a buffering agent, effectively suppressing the accumulation of OH^−^. Based on this, at a ZUR of 43% and a surface capacity of 5 mAh cm^−2^, Zn–Zn symmetric cells can cycle stably for over 350 h [128].

In addition to using single electrolyte additives, introducing a combination of multiple additives into the electrolyte has proven to be an effective strategy for improving the ZUR from multiple angles [129]. Different types of additives, through their synergistic effects, optimize the electrochemical behavior of Zn anode at different levels, thereby significantly enhancing the stability and CE of the Zn anode [130]. For instance, combining organic additives with inorganic salts can address the limitations that may arise from using a single additive, leading to more comprehensive performance improvements. For example, Wang et al. designed a mixed electrolyte based on 1,2-dimethoxyethane (DME) and I^−^ ions as two additives, which achieved stable Zn deposition/stripping at a ZUR of 75.5%. In this system, DME disrupts the hydrogen-bonding network of H_2_O and participates in the solvation structure of Zn^2+^, thereby excluding H_2_O from the Zn^2+^ solvation shell. I^−^ ions adsorb firmly on the Zn anode, lowering the desolvation energy barrier of Zn^2+^ and inducing uniform nucleation behavior, thereby contributing to stable and uniform Zn deposition and stripping [131].

### Hydrogel Electrolyte

In addition to optimizing the electrolyte by adding additives, hydrogel electrolytes have become a hot research topic as a strategy to achieve Zn anodes with high ZURs in recent years [132]. Hydrogel electrolytes are gradually being recognized as promising alternatives to liquid electrolytes due to their excellent flexibility, biocompatibility, and high ionic conductivity. They have shown great potential, particularly in fields such as wearable flexible devices [133, 134]. Well-designed hydrogel electrolytes typically contain abundant hydrophilic functional groups, which effectively convert H_2_O molecules from free water to bound water. Functional groups can further influence the dynamic interfacial environment by regulating ion flux, local water distribution, and concentration gradients near the Zn surface. This process reduces the reactivity of water molecules and minimizes side reactions caused by water, such as hydrogen evolution and corrosion [129, 135]. Moreover, the hydrogen-bonding network formed between polymer chains and water molecules in hydrogel electrolytes enables the maintenance of good mechanical properties while effectively suppressing Zn dendrite growth. Due to the flexible nature of hydrogel electrolytes, they can provide better mechanical support during charge/discharge cycling [136]. Additionally, the solid-like characteristics of hydrogel electrolytes help to enhance battery safety. Especially in large-scale applications, they significantly reduce safety hazards associated with the leakage of liquid electrolytes [137, 138].

Guo et al. reported a biocompatible hydrogel electrolyte using hyaluronic acid (HA) [121]. Due to the abundant hydrophilic functional groups in hyaluronic acid, the gel-based electrolyte provides excellent corrosion resistance for the Zn anode and regulates the nucleation and growth of Zn (Fig. 8j). Therefore, the Zn anode based on this HA gel electrolyte achieved an ACE of 99.71% and maintained a cycle life of 250 h at an 80% ZUR. In addition, cellulose-based hydrogel electrolytes (BC) have also been proven to enable stable cycling of Zn–Zn cells for 100 h at 85% ZUR with a limited electrolyte (electrolyte-to-capacity ratio *E*/*C* = 1.0 g (Ah)^−1^). Recent studies have further developed multicomponent synergistic regulation strategies to simultaneously optimize the anode interface stability and cathode redox reversibility in Zn batteries. Specifically, Zhang et al. [139] developed a biopolymer-based hydrogel electrolyte composed of sodium alginate (SA) and quaternized chitosan (qChi) for aqueous Zn||I_2_ batteries. In this system, the abundant –COO^−^ groups in the SA hydrogel matrix facilitate rapid Zn^2+^ migration and uniform Zn deposition, while the introduced qChi component helps regulate the Zn/electrolyte interface and suppress parasitic reactions. Benefiting from this synergistic interface-bulk regulation, under practical high-utilization conditions using 20 μm Zn foil, the electrolyte enabled a high ZUR of 84.6%, much higher than those of ZSO and SA-ZSO electrolytes. In addition, the Zn||Zn cell maintained stable cycling for over 1200 h at a high ZUR of 68.4%, demonstrating the effectiveness of this hydrogel electrolyte in improving Zn reversibility under high-ZUR conditions. Inspired by the above progress in biopolymer hydrogels for high-ZUR Zn anodes, advanced multicomponent hydrogel electrolytes with synergistic regulation functions have been further developed to pursue higher Zn utilization and more stable long-cycle performance.

It should be noted that the effectiveness of hydrogel electrolytes under high-ZUR conditions depends not only on their ability to reduce water activity, but also on their mechanical adaptability to repeated Zn volume changes. At extreme depths of discharge, thin Zn anodes undergo pronounced local volume fluctuation during Zn plating/stripping, which can generate cyclic interfacial stress and potentially lead to hydrogel deformation, interfacial debonding, or contact loss. Therefore, hydrogel matrices for high-ZUR Zn anodes should possess sufficient elastic recovery, fatigue resistance, and interfacial adhesion. Flexible polymer chains, dynamic reversible cross-linking networks, reinforcing fillers, and zincophilic/adhesive functional groups are useful design elements for dissipating mechanical stress and maintaining intimate Zn/electrolyte contact. Although the reported stable cycling at high ZUR indirectly suggests the feasibility of such hydrogel designs, future studies should provide more direct mechanical and interfacial evidence, such as cyclic loading–unloading tests, Zn/hydrogel adhesion measurements, in situ contact observation, post-cycling cross-sectional characterization, and impedance evolution analysis.

Beyond anode and electrolyte regulation, separator engineering also plays an important role in stabilizing Zn anodes under high-ZUR conditions, particularly by regulating ion transport and mitigating dendrite penetration. Therefore, the following section summarizes recent progress in separator design for high-ZUR ZIBs.

## Separators

As the key component separating the cathode and anode while enabling ion transport, the separator directly affects Zn^2+^ flux distribution and interfacial stability during repeated Zn plating/stripping. Compared to the extensive research on electrolytes and electrode materials, studies on cost-effective and efficient separator design strategies for achieving high ZUR in ZIBs are relatively scarce. Most of the ZIBs reported so far utilize commercially available glass fiber (GF) separators. While these GF separators are widely used, they have several limitations [140]. Firstly, they require a large amount of electrolyte to be filled, and their mechanical strength is limited, which can lead to the amplification of Zn dendrites and the puncturing of the separator [141, 142]. Notably, during the charge/discharge process, when the current density exceeds the diffusion limit of Zn^2+^, local concentration polarization forms, leading to the growth of Zn dendrites [143, 144]. These dendrites can grow along the pores of the separator and eventually pierce through the glass fiber separator, causing a short circuit [145]. Current strategies for separator design can be categorized into two approaches: the first involves modifying traditional GF separators to address their inherent shortcomings, while the second focuses on developing new separators with Zn affinity and good electrolyte wetting properties to improve the electrochemical performance of Zn anodes [146, 147]. These separator optimization strategies aim to enhance the stability and performance of the anode, prevent the formation of dendrites, and improve the overall cycling efficiency and safety of ZIBs [148].

**Fig. 9:**
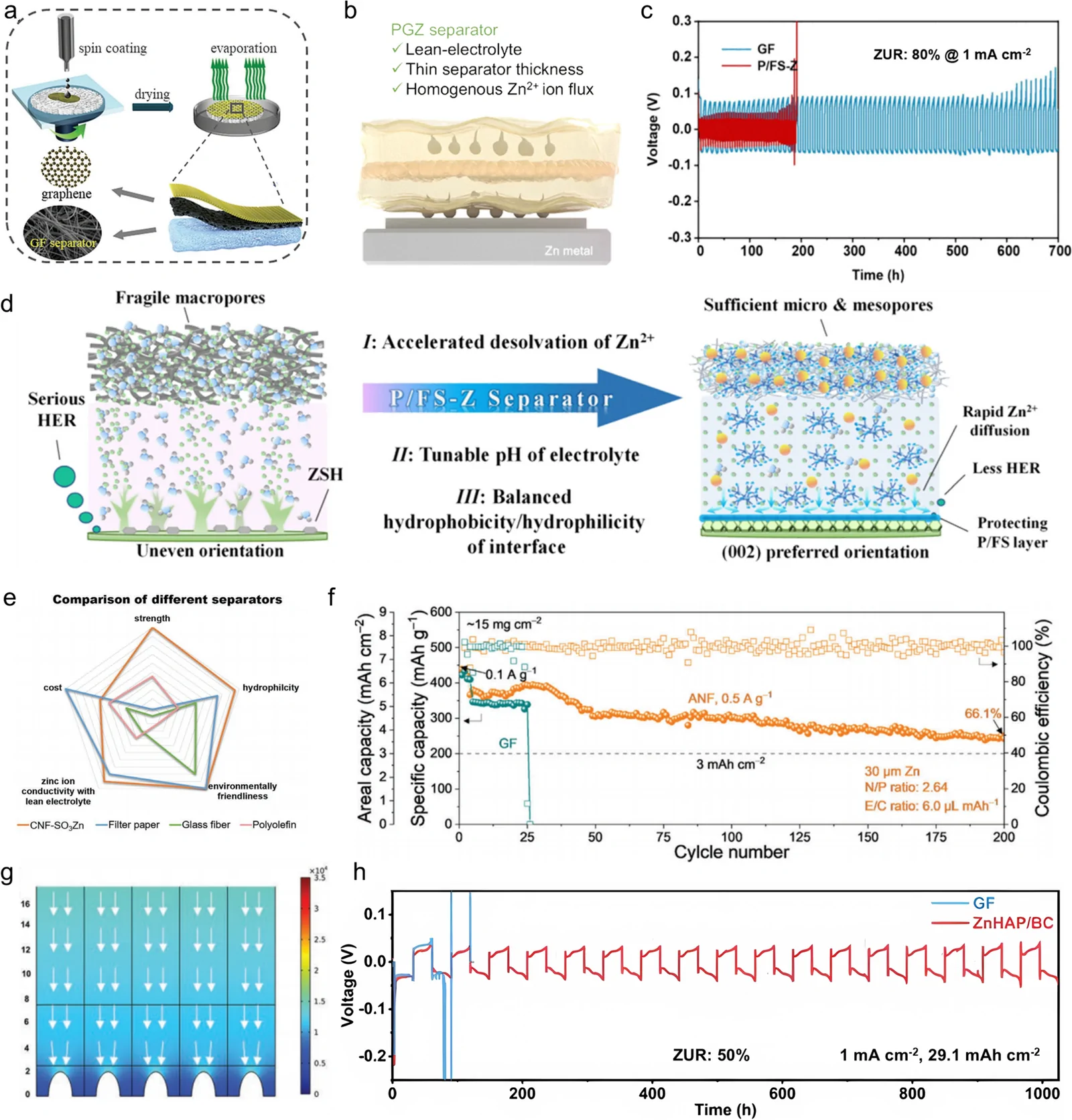
**a** Schematic illustration of the fabrication process of the Janus separator. From Ref. [61] Copyright 2022, Wiley–VCH. **b** Schematic diagram of PGZ diaphragm in reducing the amount of electrolyte and homogenizing the flux of Zn ions. From Ref. [149]. Copyright 2025, Wiley–VCH. **c** Voltage profiles of the Zn||Zn cells cycled at 80% ZUR. **d** Comparison of the interfacial reaction mechanism of the Zn anode with different separators. From Ref. [150]. Copyright 2023, Royal Society of Chemistry. **e** A performance comparison of CNF-SO_3_Zn with other separators. From Ref. [151]. Copyright 2022, Wiley–VCH. **f** Cycling performance of the Zn||MVOH (high-loading≈15 mg cm^−2^) full cells with different separators. From Ref. [62]. Copyright 2024, Wiley–VCH. **g** Electrical field simulations based on the ZnHAP/BC separator. **h** Cycling performance of Zn anodes at a high ZUR of 50%. From Ref. [152]. Copyright 2023, Wiley–VCH

Zhou and Zhu et al. [61] developed a Janus separator by spin-coating commercially available glass fiber separators with sulfonated cellulose-modified graphene sheets on one side (Janus separator). This Janus separator enables directional Zn deposition and suppresses dead Zn-induced pore clogging at high areal capacities via asymmetric interfacial electric field modulation and synergistic ion‑sieving effects. The sulfonic cellulose–graphene side provides a uniform, negatively charged interface that homogenizes the interfacial electric field, repels anions, anchors protons, and selectively transports Zn^2+^, thus eliminating tip‑induced field distortion and guiding layer‑by‑layer planar Zn (002) deposition (Fig. 9a). Meanwhile, the compact graphene layer acts as a physical barrier to block dead Zn and by-products from entering separator pores, while its robust mechanical strength withstands volume changes to preserve pore integrity. These synergistic effects fundamentally break the failure cycle of uneven deposition, dead Zn accumulation, and pore clogging, enabling stable cycling under high-areal-capacity conditions. As a result, the Zn–Zn symmetric cell with this Janus separator demonstrated over 220 h of stable cycling at a ZUR of 56%. Wu et al. [149] developed a low-cost 10 μm ultra-thin PGZ separator (*β*-PVDF/GF/Zn(OTF)_2_) for lean-electrolyte ZIBs. *β*-PVDF enables fast Zn^2+^ conduction while blocking OTF^−^, I^3−^, I^5−^, and H_2_O via electrostatic interaction and selective permeation, homogenizing Zn^2+^ flux, suppressing polyiodide shuttle and parasitic reactions; GF boosts mechanical strength (Young’s modulus: 3.7 GPa) to resist cycling-induced deformation; Zn(OTF)_2_ optimizes pore structure and electrolyte wettability. The PGZ separator delivers stable cycling for 3500 h in Zn||Zn symmetric cells (0.5 mA cm^−2^, 0.5 mAh cm^−2^), and retains a 200 h lifespan under harsh conditions (85.4% ZUR, 20 μm Zn foil, *E*/*C* = 2 μL mAh^−1^). In Zn||I_2_ full cells with high I_2_ loading (10.4 mg cm^−2^) and low *N*/*P* ratio (3.9), it achieves 2750 cycles, 93.1% capacity retention at 3C under lean electrolyte (2 μL mg^−1^), delivering 129.7 Wh kg^−1^ gravimetric energy density.

Liu et al. [150] developed a novel inorganic/organic hybrid separator using a unique wet rolling method, which consists of polytetrafluoroethylene (PTFE), gas-phase silica, and Zn-based salts (simplified as P/FS-Z). The P/FS-Z separator has three key regulatory functions: the desolvation of Zn^2+^ ions, control of the electrolyte’s pH value, and the hydrophobic/hydrophilic properties of the interface. These functionalities enhance the Zn mass transport balance and improve the controllability of interfacial reactions. The newly developed P/FS-Z separator enables long-term cycling stability, achieving 700 h of operation at a ZUR as high as 80% (Fig. 9c, d). Moreover, the high-energy–density pouch cell with a low *N*/*P* ratio (1.17) achieved an energy density of 139.9 Wh kg^−1^. Cui and Zhao et al. [151] developed a single-ion Zn^2+^ conductive nanocellulose membrane (CNF-SO_3_Zn separator) to enhance the performance of ZIBs. The separator was optimized by simultaneously improving key properties such as mechanical strength, strong hydrophilicity, uniform pore distribution, and effective Zn^2+^ ion conductivity. These improvements help mitigate the issues of HER, corrosion, and dendrite growth on the Zn anode, thus enhancing the reversibility and utilization of the Zn anode in aqueous ZIBs. With the CNF-SO_3_Zn separator, the Zn–Zn battery demonstrated a ZUR of 50% and achieved stable cycling for 100 h (Fig. 9e). Yang et al. [62] reported a novel ultra-thin aromatic polyamide nanofiber (ANF) separator with a remarkable thickness of only 5 μm. This separator is characterized by rich polar functional groups, interconnected nanopores, and high mechanical strength. The unique surface polarity of the ANF separator alters the solvation structure, promotes desolvation, and regulates the deposition direction of Zn^2+^ ions. Even at high ZURs of 50% and 80%, the Zn anode maintains an extended cycling life of more than 475 and 200 h, respectively. Furthermore, when paired with a thin Zn anode and a high-capacity Mn_2.5_V_10_O_24_·5.9H_2_O cathode in a full battery with a low *N*/*P* ratio of 2.64, the battery demonstrates excellent weight and volume energy densities of 129.2 Wh kg^−1^ and 142.5 Wh L^−1^, respectively (Fig. 9f). He et al. [152] developed a novel biodegradable separator (ZnHAP/BC) by combining natural green bacterial cellulose (BC) with nano-hydroxyapatite (HAP). This new separator effectively limits the activity of H_2_O molecules and enhances the ion transport kinetics, achieving stable reversibility and high ZUR. On the one hand, the BC substrate in ZnHAP/BC is rich in -OH functional groups, which can form hydrogen bonds with H_2_O molecules in the Zn(H_2_O)_6_^2+^. This interaction reconstructs the solvation structure of the Zn^2+^ ions, accelerates their desolvation process, and suppresses water-related side reactions on the anode surface. On the other hand, the incorporation of HAP improves the Zn^2+^ transport behavior, enhancing the ion transport dynamics, balancing the Zn^2+^ flux on the electrode surface, and reducing concentration polarization at the electrode–electrolyte interface, which promotes uniform and rapid Zn^2+^ deposition (Fig. 9g). As shown in Fig. 9h, even under 50% ZUR conditions, the Zn anode remains stable for 1000 h of cycling. Furthermore, a Zn–V_2_O_5_ battery with the ZnHAP/BC separator exhibits an impressive capacity retention of 80.5% after 1000 cycles at a current density of 1 A g^−1^ and a low *N*/*P* ratio of 2.7.

To systematically evaluate and compare the practical efficacy of the aforementioned modification strategies for enhancing high-ZUR Zn anode performance, we have compiled a table covering different classification dimensions in Table 2. Table 2 shows the comparison across the three core dimensions of battery systems, namely anode design, electrolyte optimization, and separator engineering, presenting a cross-component evaluation of the performance advantages and limitations of different modification routes under stringent practical testing conditions. As shown in these tables, all three categories of strategies have achieved remarkable progress under harsh high-ZUR conditions. Notably, multiple optimization schemes have successfully elevated the zinc utilization rate to over 80% and even exceeded 90%, while maintaining long cycling life spans ranging from hundreds to thousands of hours. This two-table comparative framework not only intuitively demonstrates the significant breakthroughs in the field of high-ZUR aqueous ZIBs and verifies the feasibility of diverse modification strategies, but also provides more comprehensive quantitative references and data support for the rational design of next-generation high-energy–density, long-cycle-life commercial aqueous zinc-ion batteries.

**Table 2:** Summary and comparison of electrochemical performance for representative Zn anodes with high utilization rates (ZURs), including various modification strategies across anodes, electrolytes, and separators under practical testing conditions

Category	Modification strategy	Areal metrics (mA cm^−2^_,_ mAh cm^−2^)	ZUR (%)	Cycling life (h)	References
Anode	Aluminum-doped zinc oxide Zn@AZO	10, 2 10, 4.69	34.1 80	600 200	[80]
	Zn_3_(BO_3_)_2_-coated Zn	50, 10	60	250	[81]
	ZnSnF@ Zn	30, 5	50	250	[82]
	Lysozyme protective layer	1, 10	77.7	1250	[83]
	Oxygen vacancy-rich CeO_2_ aerogel interface	8, 10	85	1200	[57]
	Zinc metharcylate layer	20, 20 30, 30	38.10 57.16	1153 220	[87]
	GPE-CB-ZnP	2, 9.2	96	430	[88]
	4Mpy SAMs	10, 9	90	200	[89]
	ZnS/Zn/Cu double-sided engineering	5, 5	85	300	[153]
	BN–Zn–Cu	4, 4	66	180	[56]
Electrolyte	Sulfolane additives	24, 24	96	110	[55]
	50% propylene carbonate	2.5, 10	68	160	[115]
	10 mM DMI	2, 28.4	98	550	[117]
	ZnBF/Ac-CE	6.26, 3.13	20	300	[44]
	PA	2, 50 4.4, 88.5	52.2 88.5	392 140	[116]
	6 wt% PDDA-Cl	10, 20 10, 25	68.3 85.4	700 300	[118]
	0.0085 m La(NO_3_)_3_	10, 5.93	80	160	[119]
	PMCNA	2, 11.6	80	400	[120]
	Hyaluronic acid gel	6.5, 6.5	80	250	[121]
	Colloid-6	10, 20 10, 25	68.3 85.4	700 300	[118]
Separator	PGZ separator	10, 10	85.4	200	[149]
	P/FS-Z	1, 4.68	80	700	[150]
	CNF-SO_3_Zn	1, 5	80	60	[151]
	ANF	**–**	80	200	[62]
	ZnHAP/BC	1, 29.1	50	1000	[152]
	Janus separator	28.3, 28.3	56	220	[61]
	MiB	1, 3	51.6	570	[154]
	CCN@GF	1, 10	34.2	1200	[155]
	Cu-coated	11, 11	94	200	[156]
	B/CNF	1, 30	51.24	250	[157]

## Future Perspectives

**Fig. 10:**
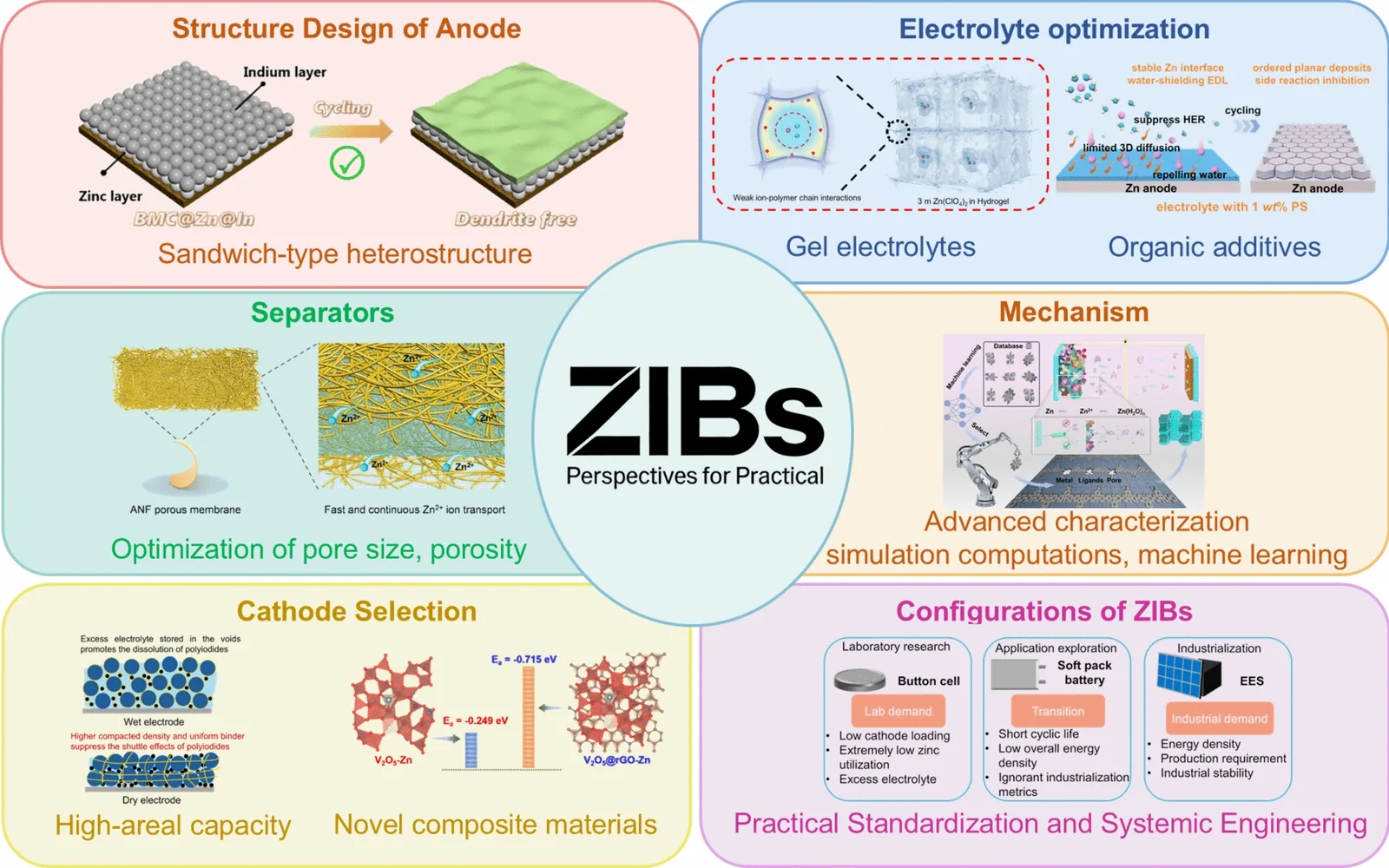
Summary of perspectives for practical ZIBs

Aqueous ZIBs have garnered widespread attention due to their unique advantages of high safety, low cost, good cycling stability, and environmental sustainability, offering strong commercialization prospects. However, ZIBs are still far from commercialization, with low ZUR remaining a major barrier to their practical application. The use of excess Zn as the anode leads to extremely low ZUR, resulting in a significant gap between the actual and theoretical energy densities of ZIBs. Therefore, developing efficient, stable ZIBs with high ZUR has become a key directions for the future commercialization. As mentioned above, we summarize research on high ZUR, thoroughly discussing the challenges in achieving high ZUR. Finally, based on current research findings and the authors’ insights, we discuss future research directions for high-utilization aqueous Zn anodes (Fig. 10), aiming to advance the future development of commercialized ZIBs.

*Structure Design of Anode:* The Zn anode with high-ZUR conditions faces side reactions such as dendrite growth, HER, corrosion, and passivation, which can severely impact the cycling performance of ZIBs. These side reactions are particularly pronounced under high-ZUR conditions and manifest in two main ways: First, dendrite growth may cause short-circuiting of the anode, leading to a sharp decline in battery performance; and second, insufficient CE may lead to the complete consumption of the Zn anode, resulting in total damage and failure of the electrode structure. Therefore, rational structure design of the anode is key to addressing the stability issues of the Zn anode under high-ZUR conditions [158]. In traditional designs, the Zn metal anode serves both as the active material for the anodic reaction and as the current collector, which subjects the Zn anode to substantial mechanical and electrochemical stress during charge/discharge cycling. This can result in electrode contact failure and material fatigue. Consequently, reducing the burden on the Zn metal is an effective approach to improving its cycling performance and stability. Specifically, in addition to building protective layers [159] on the anode surface or alloying [48] to suppress side reactions, a suitable current collector substrate can be introduced to further enhance the stability of the anode. On the one hand, a protective layer can be constructed on the front surface of ultra-thin Zn foil to prevent direct exposure of Zn metal to the electrolyte, thus reducing the occurrence of dendrite growth, hydrogen evolution, and corrosion. On the back surface of the Zn foil, a thin layer of metal (such as Cu) can be sputtered as a current collector support. Cu, being an excellent conductor, can efficiently integrate current, while its strong mechanical properties provide better structural support for the Zn metal, thereby alleviating the structural changes of the Zn anode under high-ZUR conditions. On the other hand, it is also possible to optimize pre-deposited Zn anodes and Zn powder anodes using protective layers. By carefully designing the current collector and constructing a protective layer on the anode surface, the stability of Zn anode with high ZUR can be effectively improved, extending their cycle life and advancing the practical application of ZIBs.

*Electrolyte optimization:* In aqueous electrolytes, the Zn plating/stripping process inevitably interacts with highly active H_2_O molecules, leading to corrosion and HER side reactions associated with solvent H_2_O molecules. This results in suboptimal CE, poor cycling performance, and low ZUR in aqueous electrolytes. By optimizing the electrolyte composition, such as adding inorganic additives, organic additives [160], “salt-in-water” electrolytes, and gel electrolytes [161], the electrochemical performance of the Zn anode with high ZUR can be improved. These strategies work by inducing Zn deposition on specific crystal planes, promoting Zn^2+^ desolvation, forming SEI, altering the solvation structure of Zn^2+^, and reducing the number of active water molecules. However, several potential challenges must also be considered in electrolyte optimization. For example, excessive use of additives may reduce the ionic conductivity of the electrolyte, thereby affecting the rapid plating/stripping of Zn at high current densities. Furthermore, the introduction of additives may increase the cost and weight of the electrolyte, reducing the overall energy density of the battery. Additionally, gel electrolytes, as an alternative to liquid electrolytes, can effectively reduce side reactions and improve safety, but their ionic conductivity is much lower than that of liquid electrolytes, which may impact the rate capability and power density of the battery. Moreover, the thickness of the gel electrolyte needs to be strictly controlled, as a thicker gel layer can significantly affect the battery’s energy density. Another issue is that the introduction of excessive organic components may make the electrolyte flammable, negating the inherent safety advantages of aqueous electrolytes and increasing the safety risks of the ZIBs. From a sustainability perspective, it should also be noted that although AZIBs are widely regarded as environmentally friendly and cost-effective, some recently developed high-ZUR strategies rely on fluorinated polymers, expensive organic solvents, and non-degradable hydrogel systems. These materials may introduce additional cost, environmental burden, and challenges for large-scale manufacturing and recycling, potentially offsetting the intrinsic advantages of aqueous systems. Therefore, future research should place greater emphasis on balancing electrochemical performance with material sustainability by developing greener, low-cost, and scalable components and electrolyte systems that are compatible with practical applications. Therefore, the key to achieving high-energy–density aqueous ZIBs in the future lies in designing low-concentration, low-cost, high-efficiency additives and optimizing “dilute” Zn-based electrolytes. This approach not only enhances the cycling stability and CE of the Zn anode but also maximizes the energy density and rate capability of the battery while ensuring safety and cost-effectiveness. This will be an essential pathway for advancing the commercialization of aqueous ZIBs.

*Separators:* In battery systems, separators are widely recognized as one of the key components, which not only serve as reservoirs for electrolytes and pathways for ion transport, but also provide physical separation to prevent short circuits. However, the commonly used commercial GF separators exhibit poor mechanical properties and uneven pore distribution, which makes the Zn anode prone to dendrite growth during charge/discharge cycles, accelerating puncture of the separator and ultimately leading to battery failure. Furthermore, compared to the separators used in LIBs, the thicker structure of GF separators not only reduces the energy density of the battery but also increases production costs, thereby limiting the large-scale application of aqueous ZIBs. Therefore, to achieve the next generation of ZIB separators, researchers need to innovate in several areas, particularly the optimization of pore size, porosity, and mechanical strength. The optimized separator should be capable of maintaining excellent mechanical strength and stability while further improving ion conductivity and effectively suppressing dendrite growth [62], thereby extending battery life span and increasing energy density. This will help drive the commercialization of aqueous ZIBs and enable more efficient and safer energy storage solutions.

*Advanced Characterization and Simulation:* To fully understand the specific details of failure mechanisms in high-ZUR anodes, in addition to conventional electrode and electrolyte performance verification, a combination of advanced characterization techniques is essential to deeply analyze the electrochemical behavior and failure mechanisms of the Zn anodes. Besides traditional basic analytical methods such as scanning electron microscopy (SEM), X-ray diffraction (XRD), and in situ optical microscopy, synchrotron-based comprehensive characterization methods provide more detailed insights into the microscopic mechanisms in aqueous Zn anodes with high ZUR. These advanced characterization tools can help reveal the microstructural changes that occur in the Zn anode during charge and discharge processes. In addition, in situ characterization techniques for electrodes and electrolytes, such as in situ SEM, in situ TEM, in situ Raman spectroscopy, and in situ IR spectroscopy, enable real-time monitoring of internal changes within the battery during actual cycling. These methods reveal the specific mechanisms by which the performance of high-utilization Zn anodes improves. Furthermore, density functional theory (DFT) calculations and molecular dynamics (MD) simulations provide theoretical support for studying the failure mechanisms of high-ZUR anodes and improving their stability. By simulating the Zn deposition/stripping process under different electrolyte compositions, anode materials, and working conditions, we can better understand the key factors that influence the performance of Zn anodes. Beyond traditional experimental methods, high-throughput screening and data-driven machine learning approaches have also introduced new strategies for rapid optimization of materials and electrolytes for ZIBs. By integrating deep learning and other artificial intelligence (AI) tools, we can more efficiently select the best-performing electrode structures and electrolyte compositions from a large pool of materials [162]. Constructing appropriate descriptors and datasets and leveraging these advanced productivity tools can significantly accelerate the optimization process and improve screening efficiency. By combining advanced characterization techniques, simulation computations, and data-driven optimization methods, we can gain a more comprehensive understanding of the failure mechanisms of high-ZUR anodes. This provides new avenues for improving the stability and utilization of aqueous ZIBs. In particular, with the aid of in situ characterization technologies and AI-assisted screening, material development efficiency can be significantly enhanced, thus accelerating the optimization process of aqueous ZIBs. This will lay the foundation for the commercialization of high-energy–density, high-cycle-stability aqueous ZIBs.

*Cathode Selection:* To build high energy density and stable aqueous ZIBs, the selection of cathode materials is as crucial as improving the utilization of the Zn anode. Currently, there are various options for cathode materials in aqueous ZIBs, each with its unique advantages and limitations. Oxide-based materials are cost-effective and stable, but they suffer from poor conductivity and rapid capacity degradation; Organic materials are environmentally friendly and highly tunable, but they often exhibit poor cycling stability. Manganese-based materials are low-cost and offer relatively high energy densities, but they have lower stability and capacity retention. Carbon-based materials are stable and cost-effective, but their voltage platform and specific capacity are low, which limits the overall energy density. It is noteworthy that most of the reported cathode materials for aqueous ZIBs face challenges in achieving both high areal capacities and long cycling stability. This issue remains a significant bottleneck preventing aqueous ZIBs from reaching high energy density. To address this problem, future research may need to focus on the following areas: Development of high-areal capacity cathode materials: Emerging materials, such as halogen-based batteries (e.g., chlorine, bromine, iodine), show potential [163]. Halogen materials can offer higher theoretical capacities, potentially leading to higher energy densities. However, one of the main challenges in their practical application is corrosion. Therefore, designing effective electrolytes or interface protective layers to prevent the corrosion of halogen materials remains a critical challenge that needs to be addressed. Optimization of existing materials: In addition to exploring new material systems, further optimization of the performance of existing materials is key to improving the energy density of aqueous ZIBs. Strategies such as nanostructuring and doping can improve the conductivity, stability, and cycling life of the materials. To overcome the limitations of single-material cathodes, the development of composite cathode materials has become an important research direction. Composite materials can combine the advantages of different materials, improving overall performance [164]. Reasonably designing the electrolyte composition and optimizing the interface reactions between the electrolyte and cathode material can significantly improve the stability and cycling performance of the cathode. For example, using solvents with lower corrosivity or adding surfactants to improve the interface layer can reduce side reactions and enhance the cycling efficiency and life span of the battery. In summary, future research will focus on optimizing the performance of existing materials, developing novel composite materials, and improving cathode material performance through rational design of electrolytes and battery structures. This will lay the foundation for the commercialization of aqueous ZIBs with high energy density.

*Configurations and Manufacture of ZIBs for Practical Applications* As aqueous ZIBs are scaled up from laboratory experiments to practical applications, the structural configuration of the full battery becomes a key factor influencing its operational performance. While performance tests in laboratory settings typically use coin cells, practical applications require a wider variety of battery types, such as prismatic cells and pouch cells. Different battery structures can have distinct effects on parameters such as operational efficiency, capacity density, and thermal management. In particular, pouch cells are more relevant for evaluating the practical feasibility of high-ZUR ZIBs because they involve larger electrode areas, stacked or laminated configurations, and more realistic electrolyte distribution than coin cells. Under high-ZUR conditions, the limited Zn reservoir makes pouch cells more sensitive to non-uniform current distribution, local Zn depletion, electrode deformation, gas generation, and electrolyte starvation. These issues may be less obvious in small coin cells but can be amplified during scale-up. Therefore, future studies should pay more attention to high-ZUR pouch cells under practical conditions, including low *N*/*P* ratios, lean electrolyte, high cathode loading, and long-term cycling, to better assess the reliability and commercialization potential of aqueous ZIBs. Therefore, future research should focus on optimizing different battery types and exploring designs that are more suitable for large-scale production and practical deployment. In addition, current laboratory testing conditions are often mild, and many tests involve excess Zn, electrolytes, and relatively low cathode loads, which can exaggerate the electrochemical performance of the ZIBs. For example, excess Zn anodes, excess electrolytes, and lower cathode loadings are often used to highlight the advantages of zinc-ion batteries. However, this idealized testing environment does not fully reflect the real-world conditions the battery may encounter. Thus, future testing should more rigorously simulate actual operating environments, including strict control of the anode/cathode (*N*/*P*) ratio from a practical application perspective, optimizing the electrolyte ratio, and avoiding excessive Zn or electrolyte use. Additionally, the electrolyte/capacity (*E*/*C*) ratio represents another decisive factor for practical high-ZUR ZIBs. A low *E*/*C* ratio (lean electrolyte) is indispensable for achieving high practical energy density, yet it imposes stringent constraints on long-cycle stability. Under lean-electrolyte conditions, the limited electrolyte volume cannot sustain the continuous repair of SEI damaged by persistent side reactions such as hydrogen evolution, corrosion, and passivation at the anode/electrolyte interface. Electrolyte depletion rapidly disturbs Zn^2+^ transport and homogenization, while the finite Zn source under high ZUR suffers irreversible consumption from dendrite growth and dead Zn formation. This synergistic “dual depletion” of electrolyte and Zn jointly triggers a sharp rise in electrode polarization and premature battery failure, which is far more detrimental than the individual depletion of Zn or electrolyte under excess electrolyte conditions. This coupled dual depletion remains a major unresolved challenge for practical long-cycle ZIBs. Although current strategies including anode protective layers, electrolyte optimization, and functional separators can alleviate dual depletion by stabilizing interfaces, reducing free water activity, and homogenizing Zn deposition, the field still lacks robust anti-decomposition interphase designs, standardized low-*E*/*C* testing protocols, and clear mechanistic understanding of the Zn–electrolyte depletion coupling behavior [139, 161]. Therefore, standardized low *E*/*C* ratios must be universally adopted in laboratory evaluations to faithfully reflect the real performance and feasibility of high-ZUR Zn anodes toward industrial applications. This will provide a more accurate assessment of the battery’s stability and efficiency over long-term usage. In the design of battery systems, the optimization of both active materials and non-active components (such as current collectors, casings, etc.) plays a crucial role in the overall performance of the battery. Optimizing these non-active components, particularly by using lighter, more conductive, and corrosion-resistant materials, can significantly enhance the energy density and reduce both the weight and cost of the battery. Finally, considering that batteries may encounter extreme conditions during practical use, such as high or low temperatures, varying humidity, or prolonged high-load usage, conducting electrochemical performance tests under these extreme conditions may provide valuable insights for optimizing battery design. More importantly, wide temperature fluctuations should receive particular attention for high-ZUR Zn anodes. Under low-temperature conditions, the reduced ionic conductivity and sluggish Zn^2+^ desolvation kinetics can intensify concentration polarization, leading to uneven Zn deposition and accelerated Zn depletion. In contrast, elevated temperatures may aggravate HER, corrosion, and passivation, thereby lowering CE and consuming the limited Zn inventory more rapidly. These effects are especially detrimental under high-ZUR conditions, where only a small excess of Zn is available and even minor irreversible Zn loss can trigger rapid capacity decay or electrode failure [22]. Therefore, future studies should evaluate high-ZUR Zn anodes under realistic thermal cycling conditions, such as repeated low-/room-/high-temperature transitions, rather than only at a fixed temperature. Such tests would better reflect practical operating environments and provide a more reliable assessment of interfacial stability, Zn reversibility, and long-term durability. Simulating these harsh environments can reveal the stability, life span, and safety of the battery under challenging conditions, providing essential technical assurance for commercialization [165]. Such research will lay the foundation for the commercialization of safe, cost-effective, high-energy–density aqueous ZIBs with excellent environmental adaptability, paving the way for their broad deployment in practical applications [166].

In conclusion, significant progress has been made in the development of high-ZUR ZIBs. Achieving the widespread use of ZIBs requires substantial research and innovation. Future development must address not only the performance bottlenecks of Zn anode and cathode materials but also optimize several aspects such as battery structure design, electrolyte formulation, and manufacturing processes. With continued advances in mechanism research and the emergence of new materials and technologies, it is anticipated that high ZUR, long-cycle stability, and high energy density in aqueous ZIBs will be realized for commercial applications. We are confident that, with the collective efforts of the global scientific community and ongoing technological innovations, aqueous ZIBs will play an increasingly crucial role in energy storage systems. They will emerge as strong candidates for next-generation, efficient, safe, and cost-effective energy storage solutions.
